# Ferromagnetic superconductivity with excitonic Cooper pairs: Application to Γ-valley twisted semiconductors

**DOI:** 10.1126/sciadv.aeb4888

**Published:** 2026-05-20

**Authors:** Daniele Guerci, Liang Fu

**Affiliations:** Department of Physics, Massachusetts Institute of Technology, Cambridge, MA 02139, USA.

## Abstract

We present a theory of ferromagnetic superconductivity that emerges upon doping a correlated ferromagnetic insulator through the condensation of excitonic Cooper pairs, which are charge-2*e* bosonic quasiparticles made of Cooper pairs strongly hybridized with excitons. Using a strong-coupling expansion supported by exact diagonalization, we analyze a model of spin-polarized electrons and show that excitonic Cooper pairs form from electron-hole fluctuations upon doping a strongly correlated insulator. We characterize their binding energy, effective mass, and the resulting superconducting transition temperature. We propose possible realization of spin-polarized superconductivity in twisted semiconductors with honeycomb moiré superlattice.

## Teaser

Doping a correlated ferromagnetic insulator induces superconductivity via condensation of excitonic Cooper pairs.

## INTRODUCTION

The search for high-temperature superconductivity driven by electron repulsion has long fascinated researchers due to its potential technological applications and fundamental scientific interest. Since the pioneering work of Kohn and Luttinger (*1*), superconductivity has been theoretically obtained from repulsive interactions in Fermi liquids, where the effective attraction arises from the oscillatory component of the screened interaction. As the Kohn-Luttinger–type theories are based on interaction expansion (*2*–*7*), it only yields weak-coupling superconductivity, whose transition temperature *T*_c_ is orders of magnitude smaller than the Fermi energy and whose coherence length far exceeds the interparticle distance.

Recently, a novel mechanism for superconductivity from repulsive interaction has been introduced for multiband systems (*8*, *9*). For simple models of correlated band insulators, it has been shown rigorously that an effective attraction between doped electrons can arise from interband charge fluctuations. These fluctuations mediating superconductivity are associated with the “vibrations” of the valence electrons (i.e., excitons) (*10*), as opposed to the ion lattice vibrations (i.e., phonons) in conventional superconductors. Possible applications of this electronic pairing mechanism have been discussed for various models and materials (*11*–*19*).

In this work, we extend this framework to develop a theory of ferromagnetic superconductivity that arises from doping a strongly correlated ferromagnetic insulator. This unconventional superconducting state is spontaneously fully spin-polarized and features tightly bound electron pairs dressed with excitons. We focus on a regime where charge fluctuations remain dynamical and cannot be integrated out, leading to bound states with qualitatively distinct dynamical properties beyond those considered in earlier works (*8*, *9*). By solving a minimal model of strongly interacting electrons on the honeycomb lattice, we show explicitly that two important energy scales for superconductivity, the pairing gap and the superfluid stiffness, are both controlled by the interaction strength in our system. At optimal doping, the maximum superconducting transition temperature *T*_c_ reaches a significant fraction of the bandwidth.

**Fig. 1.**
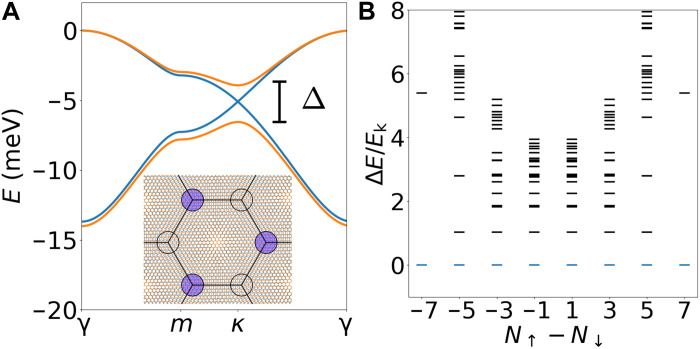
Γ-valley twisted semiconductors. (**A**) The low-energy bands, with a tunable gap Δ controlled by the displacement field *D*. Blue and orange denote *D* = 0 and *D* = 60 meV, respectively. The inset displays the moiré pattern with high-symmetry stackings (MM, MX, and XM). Wannier orbitals localize at MX and XM, forming a honeycomb lattice. (**B**) The many-body spectrum in units of *E*_k_ = *ħ*^2^/(2*ma*^2^) across different spin *S^z^* sectors for a 3 × 3 cluster, including the two topmost bands. The calculations are performed with ϵ = 10, *d*_sc_ = 5 nm and θ = 2°.

Our theory is motivated by Γ-valley twisted transition metal dichacogenides (*t*TMDs) (*20*–*23*), where Wannier orbitals are centered at the MX and XM moiré sites forming a honeycomb lattice (Fig. 1A, inset). At small twist angle, the low-energy moiré bands exhibit Dirac points similar to graphene, but have a very narrow bandwidth (Fig. 1A) suitable for strongly correlated phenomena. A recent experiment (*24*) has observed correlated insulators at the filling of *v* = 1 hole per unit cell. This opens the door to novel phenomena that remain largely unexplored compared to the more intensively studied *K*-valley TMDs (*25*–*27*).

## RESULTS

### Ferromagnetism

Γ-valley moiré semiconductors have negligible spin-orbit coupling (*28*), which leads to spin SU(2) symmetry (*20*, *21*). By exact diagonalization (ED) of the interacting continuum model for v-valley *t*TMDs, detailed in the Supplementary Materials, we find robust ferromagnetism over a wide range of twist angles and interaction strengths, both at filling *v* = 1 and under finite hole doping. Notably, these ferromagnetic ground states are fully spin polarized, having (2*S* + 1)-fold degeneracy with *S* = *N*/2 (*N* is the total number of spin-^1^/_2_ electrons). This behavior is especially pronounced near *v* = 1, as illustrated in Fig. 1B, which shows the many-body spectrum across different total spin *S^z^* sectors for *v* = 7/9. In addition, we observe (2*S* − 1)-fold degeneracy in low-lying excited states, consistent with one-magnon excitation. We also note that ferromagnetism extends to *v* = 1, where strong electron interaction induces a correlated ferromagnetic insulator with broken sublattice symmetry. This tendency is consistent with the effective spin model derived in the strong-interaction limit, as discussed in the Supplementary Materials; see also (*29*–*31*). Moreover, ferromagnetism can be further stabilized at finite doping through a Nagaoka-type mechanism (*32*).

### Extended Hubbard model on honeycomb lattice

Building on our continuum model results, we study a minimal model of spin-polarized fermions on a honeycomb lattice, incorporating the shortest-range nontrivial repulsive interactions

$$H=-t\sum_{\langle r,r^{\prime }\rangle }f_{r}^{†}f_{r^{\prime }}+V\sum_{\langle r,r^{\prime }\rangle }n_{r}n_{r^{\prime }}+\Delta N_{B}$$ (1)

where $\langle r,r^{\prime }\rangle$ denotes nearest-neighbor (n.n.) sites on the honeycomb lattice and *N*_*A*,*B*_ denotes the total number of particles on *A* or *B* sublattice, which corresponds to MX and XM moiré sites, respectively. Δ represents the potential difference between the two sublattices, which is induced by an applied displacement field *D*, as shown in the Supplementary Materials.

**Fig. 2.**
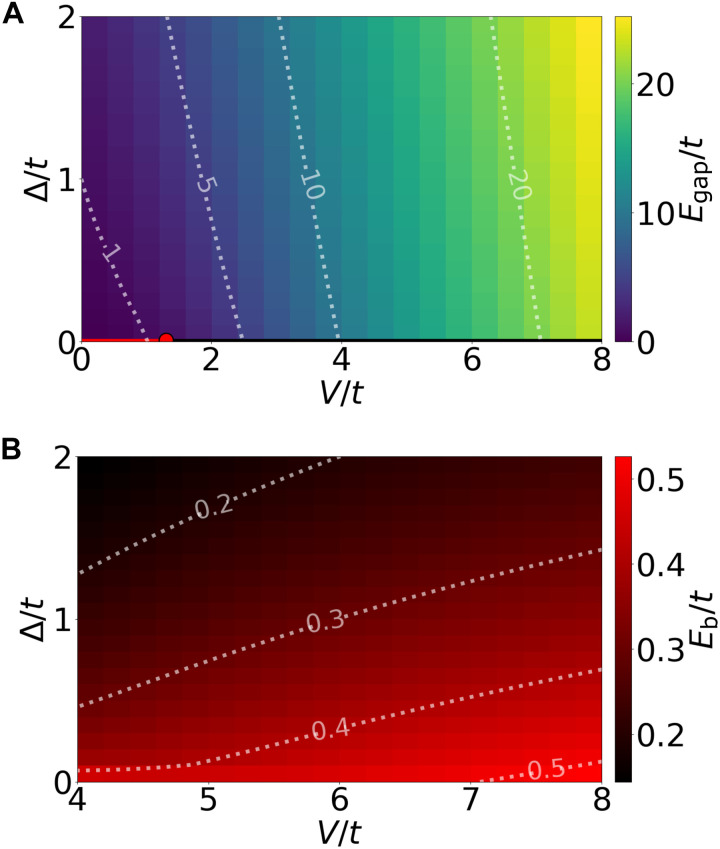
Phase diagram at *v* = 1 and finite doping. (**A**) The charge gap at *v* = 1. The red dot at ∆=0 marks the critical interaction *V*_c_ = 1.3 *t*, separating the Dirac semimetal from the sublattice-polarized insulator. (**B**) The binding energy of excitonic Cooper pairs. ED is performed on a 24-site lattice with periodic boundary conditions.

For large *V*/*t*, the ground state at *v* = 1 is a gapped insulator. The charge gap is defined as $E_{\text{gap}}=E_{+1}+E_{-1}-2E_{0}$, where *E*_0_ and *E*_±1_ denote the ground-state energies at *v* = 1 and with one additional or one removed charge, respectively. The resulting gap obtained from ED is shown in Fig. 2A. Depending on the sign of Δ, either *A* or *B* sites are preferentially occupied, while, at Δ = 0, the system spontaneously breaks the sublattice symmetry (*33*–*37*) at *V* > *V*_c_ = 1.3*t*, consistent with previous studies (*38*–*42*). In this work, we will focus on the strongly interacting regime *V*/*t* ≥ 5 where the correlation length is short, which justifies our strong-coupling expansion in *t*/*V* and mitigates finite-size effect in our ED study.

### Excitonic Cooper pair

To find the ground state at small doping *v* = 1 + δ, we analyze the energy cost of various charge-*e*, 2*e*, and 4*e* excitations of the *v* = 1 correlated insulator. The model is particle-hole symmetric, showing identical behavior for electron doping (δ > 0) and hole doping (δ < 0). Before presenting the full theory, we summarize our first main finding in Fig. 2B: the binding energy *E*_b_ of two doped particles. Within our definition, a positive binding energy (*E*_b_ > 0) signals the formation of excitonic Cooper pairs.

To understand the origin of pairing from repulsive interaction in our model, let us first consider charge excitations in the infinite coupling limit *V* → ∞. Here, the ground state at *v* = 1 is fully sublattice polarized, and quantum fluctuation is completely suppressed because any hopping process entails interaction energy cost *V*. Assuming that *A* sites are occupied at *v* = 1, adding a single particle to a *B* site costs energy $E_{1e}=\Delta +3V$ where the energy of a charge ±1*e* excitation is $E_{\pm 1e}=E_{\pm 1}-E_{0}$. This charge-*e* particle is also “frozen” because moving it costs additional interaction energy.

**Fig. 3.**
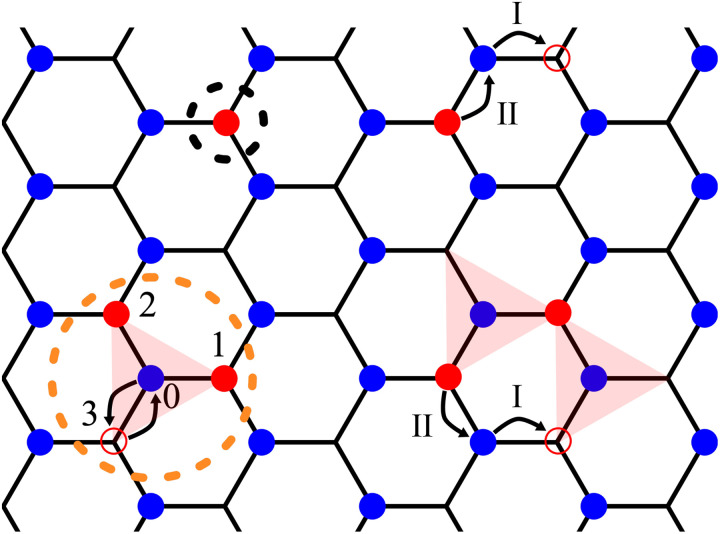
Excitonic Cooper pair and charge carrier motion. (**Left**) Charge-*e* and 2*e* excitations, highlighted by dashed circles, correspond to a fermion (black) and an excitonic Cooper pair (orange). The latter is formed as a bound state between 1 and 2 mediated by the charge-transfer exciton 0 to 3. (**Right**) Leading-order processes contributing to quasiparticle mass: (I) polaron formation and (II) its recombination, inducing center-of-mass motion.

On the other hand, consider a pair of particles added to two neighboring *B* sites, denoted as 1 and 2 in Fig. 3. This configuration is connected by the hopping term $f_{3}^{†}f_{0}$ to a “trimer” configuration, with a cluster of three particles on *B* sites 1, 2, and 3 surrounding an empty *A* site 0 (*8*). The trimer configuration costs the same interaction energy 6*V* as the initial configuration. Therefore, even at *V* → ∞, quantum hopping *t* leads to a linear superposition between a localized pair of particles and a trimer; the latter is dressed by a charge-transfer exciton ($f_{3}^{†}f_{0}$) as shown in Fig. 3. Because coherent superposition lowers the energy, the resulting charge-2*e* complex, which we call “excitonic Cooper pair,” is lower in energy than two separate charge-*e* particles.

In the *V* → ∞ limit, an isolated excitonic Cooper pair cannot move because any hopping term will only connect it to configurations that cost additional interaction energy *V*. This allows us to determine its binding energy per particle $E_{b}\equiv E_{1e}-E_{2e}/2$, where $E_{2e}=E_{+2}-E_{0}$ is the charge-2*e* excitation energy, exactly by solving our model (Eq. 1) on a four-site cluster, yielding

$$E_{b}=\sqrt{\Delta^{2}/16+3t^{2}/4}-\Delta /4$$ (2)

For any Δ/*t*, this result provides an upper bound on the charge-2*e* binding energy at finite V/*t*, as demonstrated in the Supplementary Materials. The many-body wave function of the system having a single excitonic Cooper pair centered at *A* site *r* is

$$\begin{array}{c} |\Phi_{2}\left(r\right)\rangle =\left(\frac{\alpha }{\sqrt{3}}\sum_{j=1}^{3}f_{r_{j}^{\prime }}^{†}f_{r_{j+1}^{\prime }}^{†}+\sqrt{1-\alpha^{2}}f_{r_{1}^{\prime }}^{†}f_{r_{2}^{\prime }}^{†}f_{r_{3}^{\prime }}^{†}f_{r}\right)|\Phi_{0}\rangle \\ \equiv b_{r}^{†}|\Phi_{0}\rangle \end{array}$$ (3)

where $|\Phi_{0}\rangle =\prod_{r\in A}f_{r}^{†}|0\rangle$ is the undoped ground state; $r_{j}^{\prime }$ with *j* = 1, 2, and 3 denotes the three *B* sites adjacent to r. The excitonic Cooper pair wave function resonates between a Cooper pair and a trimer, displayed in Fig. 3, with probability α^2^ and 1 − α^2^, respectively, where α depends on Δ/*t*, $\alpha^{2}=1/2+\Delta /\sqrt{4\Delta^{2}+48t^{2}}$. The pair wave function belongs to the *A*_2_ irrep of *C*_3*v*_, exhibiting *f*-wave symmetry.

Our strong-coupling result at V≫t complements the previous study in a different regime Δ≫t (*9*). Our result shows that, for large repulsive interaction, the Cooper pair is strongly hybridized with the exciton at small Δ, resulting in a large pair binding energy which reaches the maximum value ${E_{b}|}_{\Delta =0}=\sqrt{3}t/2$ at Δ = 0. As Δ increases, the hybridization with the exciton is reduced; the pair binding energy decreases monotonously and becomes vanishingly small $E_{b}\approx 3t^{2}/\Delta$ in the limit Δ≫t in agreement with (*9*). In the rest of this work, we focus on the regime of large *V*/*t* and small Δ /*t*, where the exciton binds two doped particles tightly together.

Next, we perform a strong-coupling expansion in the small parameter *t*/*V* to study the regime of large but finite interaction strength. The strong coupling expansion is performed by organizing the Hilbert space into sectors having different numbers (*M*) of n.n. occupied sites. The Hamiltonian, when decomposed into these sectors, consists of a block diagonal term and an off-diagonal term, given by $H=H_{0}+H^{\prime }$. *H*_0_ is expressed as $H_{0}=\sum_{M}H_{M}$

$$H_{M}=-t{\mathbb{P}}_{M}\sum_{\langle r,r^{\prime }\rangle }f_{r}^{†}f_{r^{\prime }}{\mathbb{P}}_{M}+\Delta N_{B}+\mathrm{MV}$$ (4)

Here, ${\mathbb{P}}_{M}$ is the projector onto the sector with *M* n.n. occupied sites, and the term *MV* is the interaction energy. The off-diagonal part, which couples sectors with different values of *M*, is given by $H^{\prime }=\sum_{M}\sum_{q\neq 0}T_{q,M}$

$$T_{q,M}=-t{\mathbb{P}}_{M+q}\sum_{\langle r,r^{\prime }\rangle }f_{r}^{†}f_{r^{\prime }}{\mathbb{P}}_{M}$$ (5)

where *T*_*q*,*M*_ changes the number of n.n. occupied sites by *q* = ±1 and ±2 in a sector with fixed *M*.

In the absence of *H*′ (or *V* → ∞), the ground states of *H*_0_ with zero (*p* = 0), one (*p* = 1), and two (*p* = 2) doped particles have a fixed number of n.n. occupied sites *M* = *zp*, leading to ground state energies $E_{p}=\mathrm{zpV}$ with *z* = 3 coordination of the lattice. Note that, in the presence of doped particles (*p* = 1 and 2), the ground states of *H*_0_ are extensively degenerate as discussed above. This degeneracy is lifted by virtual processes induced by *H*′, which couple the low-energy sector to high-energy sectors with M=zp+q costing additional interaction energies *qV*. These virtual processes are accounted for using the Schrieffer-Wolff (SW) transformation (*43*–*45*), a unitary transformation that systematically eliminates the coupling between low- and high-energy sectors: $H=e^{S}{\mathrm{He}}^{-S}$, where *S* is anti-Hermitian. The SW transformation can be carried out by a perturbative expansion in t/V: $S=S_{1}+S_{2}+\ldots$ with $S_{j}∼{\left(t/V\right)}^{j}$.

As detailed in the Supplementary Materials, we calculate *S* up to the second order (*t*/*V*)^2^, so that low- and high-energy sectors are decoupled in the transformed Hamiltonian H up to the order (*t*/*V*)^2^. Projecting H onto the low-energy manifold with *M* = *zp* yields the effective Hamiltonian of interest $H^{\left(p\right)}$, with *p* = 0, 1, and 2 denoting the number of doped particles. $H^{\left(p\right)}$ takes a particularly simple form at Δ = 0

$$H^{\left(p\right)}=H_{\mathrm{zp}}-\sum_{q=1}^{2}\frac{T_{q,\mathrm{zp}}^{†}T_{q,\mathrm{zp}}}{\mathrm{qV}}+\sum_{q=1}^{2}\frac{T_{q,\mathrm{zp}}^{†}T_{0,\mathrm{zp}+q}T_{q,\mathrm{zp}}}{{\left(\mathrm{qV}\right)}^{2}}-\frac{1}{2}\sum_{q=1}^{2}\frac{\left\{T_{q,\mathrm{zp}}^{†}T_{q,\mathrm{zp}},T_{0,\mathrm{zp}}\right\}}{{\left(\mathrm{qV}\right)}^{2}}$$ (6)

where {·,·} is the anticommutator.

We now analyze the consequences of the above strong-coupling expansion for the undoped ground state and charge excitations. For the undoped case, the effective Hamiltonian $H^{\left(0\right)}$ yields the correction to the ground state energy $\delta E_{0}=-{\mathrm{Nzt}}^{2}/\left(2V\right)$ up to $O\left(t^{4}/V^{3}\right)$, where *N* is the number of unit cells.

For one doped charge (*p* = 1), the perturbation due to *H*′ in the effective Hamiltonian lifts the degeneracy of charge-*e* excitations and endows them with an energy-momentum dispersion. This is derived by projecting $H^{\left(1\right)}$ in the degenerate manifold of unperturbed ground states $|\Phi_{1}\left(r\right)\rangle =f_{r}^{†}|\Phi_{0}\rangle$. The resulting hopping Hamiltonian for a charge-*e* quasiparticle takes the form: $H^{\left(1\right)}=E_{1}+t_{f}\sum_{\langle r,r^{\prime }\rangle \in B}f_{r}^{†}f_{r^{\prime }}$, where $t_{f}=t^{2}/V+O\left(t^{4}/V^{3}\right)$ represents the hopping amplitude between adjacent B sites illustrated in Fig. 3, and the constant energy term $E_{1}=\mathrm{zV}+\delta E_{1}$ with $\delta E_{1}=\delta E_{0}-3{\mathrm{zt}}^{2}/\left(2V\right)$ and δ*E*_0_ includes the energy correction arising from virtual processes. The corresponding dispersion relation is given by $\epsilon_{f}\left(k\right)=E_{1}+2t_{f}\sum_{j}\text{cos}\left(k\cdot a_{j}\right)$.

**Fig. 4.**
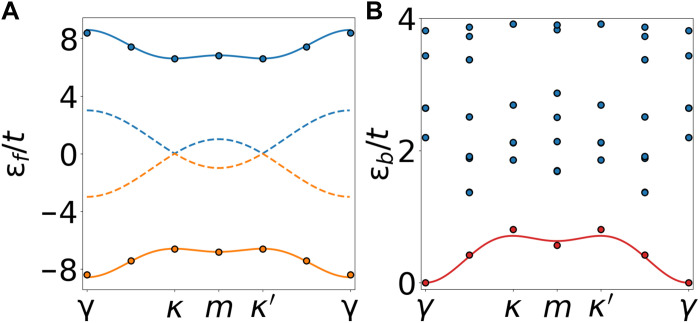
Charge-*e* and excitonic Cooper pair dispersion relation. (**A**) The ±*e* excitation spectra with (solid) and without (dashed) interactions. (**B**) The charge-2*e* quasiparticle dispersion relation. Dots: ED spectrum; solid lines: strong-coupling theory (no fit parameters). We used (Δ, *V*)/*t* = (0, 5) on a 24-site cluster.

Figure 4A shows the band dispersion of charge-1*e* excitations of the sublattice polarized insulator at *V*/*t* = 5. Results obtained from ED (dots) and strong-coupling expansion (solid lines) are found to be in excellent agreement. For comparison and contrast, we also included the bare dispersion relation (dashed lines), which features Dirac cones.

In the charge-2*e* sector (*q* = 2), excitonic Cooper pairs located at different *A* sites $|\Phi_{2}\left(r\right)\rangle =b_{r}^{†}|\Phi_{0}\rangle$ (*3*) are degenerate in the absence of *H*′ and form an orthonormal basis $\langle \Phi_{2}\left(r\right)|\Phi_{2}\left(r^{\prime }\right)\rangle =\delta_{rr^{\prime }}$. After including perturbative corrections to second order in *t*/*V*, we obtain an effective Hamiltonian within this degenerate subspace which governs the hopping of excitonic Cooper pair

$$H^{\left(2\right)}=E_{2}-t_{b}\sum_{\langle r,r^{\prime }\rangle \in A}b_{r}^{†}b_{r^{\prime }}$$ (7)

and the corresponding energy dispersion is given by $\epsilon_{b}\left(q\right)=\epsilon_{b}\left(q\right)=E_{2}-2t_{b}\sum_{j=1}^{3}\text{cos}\left(q\cdot a_{j}\right)$, where q is the Cooper pair momentum. Here, $E_{2}=2\Delta +2\mathrm{zV}-2E_{b}+\delta E_{2}$ includes correction $\delta E_{2}=\delta E_{0}-{\mathrm{zt}}^{2}/V-5\sqrt{3}t^{3}/\left(4V^{2}\right)$ due to virtual processes, which will affect the binding energy to be discussed later. The hopping amplitude of excitonic Cooper pair *t_b_* (*7*), for Δ = 0, is given by

$$t_{b}=\frac{t^{2}}{6V}+\frac{\sqrt{3}t^{3}}{2V^{2}}+O\left(\frac{t^{4}}{V^{3}}\right)$$ (8)

Here, the leading order contribution $∼t^{2}/V$ originates from the second-order particle hopping process illustrated in Fig. 3. Equation 8 also includes the next leading order contribution $∼t^{3}/V^{2}$, which originates from various third-order hopping processes as detailed in the Supplementary Materials.

Figure 4B shows the energy dispersion of charge-2*e* excitations. Results obtained from ED (dots) and our analytical expression (solid line) with *t_b_* given in Eq. 8 are found to be in excellent agreement. While the charge-*e* fermion dispersion has degenerate minima at *K*, *K*′, the charge-2*e* boson dispersion has the minimum at Γ, i.e., q=0. Comparing the energy difference between the ground states of our system doped with one and two particles, we determine the binding energy up to order $O\left(t^{3}/V^{2}\right)$, which for Δ = 0, is given by

$$E_{b}=\frac{\sqrt{3}}{2}t-\frac{3t^{2}}{V}+\frac{5\sqrt{3}t^{3}}{8V^{2}}+O\left(\frac{t^{4}}{V^{3}}\right)$$ (9)

Compared to *V* = ∞, the binding energy decreases monotonously as *V* is reduced but remains large *E*_b_ ≈ 0.31 *t* at *V*/*t* = 5.

We emphasize that all analytical results including binding energy and charge-2*e* dispersion are obtained from strong-coupling expansion to second order in *t*/*V* without any adjustable parameter. It is remarkable that analytical and ED results are in excellent agreement up to *t*/*V* = 0.2. We further extend analytical calculations to include finite Δ in the Supplementary Materials.

**Fig. 5.**
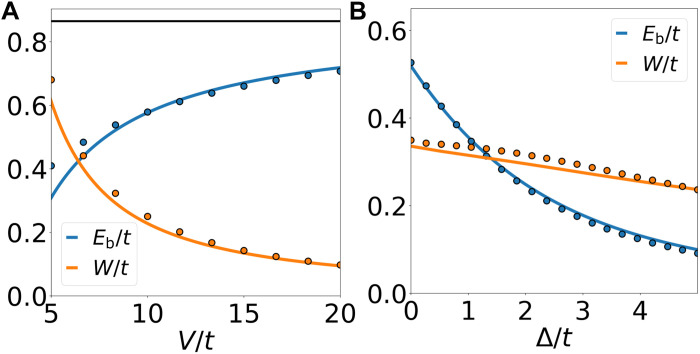
Excitonic Cooper pair binding energy and bandwidth. (**A**) *E*_b_ and *W* as a function of *V*/*t*, respectively. The solid black line shows the asymptotic value $\sqrt{3}t/2$ reached for *V*/*t* = ∞. (**B**) The evolution of *E*_b_ and *W* at *V*/*t* = 8 as a function of Δ/*t*. Twenty-four-site cluster ED (dots) and strong-coupling theory (solid lines) without any adjustable parameters.

Figure 5 summarizes the main results on the behavior of excitonic Cooper pair in our system. Figure 5A shows the evolution of the binding energy *E*_b_ and the charge-2*e* boson bandwidth *W* as a function of the interaction strength *V* for Δ = 0. As *V* increases, the binding energy *E*_b_ increases and eventually saturates, indicating the electronic origin of pairing from repulsion, while the boson bandwidth *W* decreases because virtual processes entailed in boson hopping are energetically suppressed.

Figure 5B shows the effect of Δ on *E*_b_ and *W*. The binding energy *E*_b_ in Eq. 2 is reduced by increasing Δ, which suppresses the hybridization of Cooper pair with exciton. On the other hand, at finite Δ, the bosonic hopping amplitude takes the form

$$t_{b}=\frac{t^{2}}{6\left(\Delta +V\right)}\left[1+\frac{\Delta }{\sqrt{\Delta^{2}+12t^{2}}}\right]+O\left(\frac{t^{3}}{V^{2}}\right)$$ (10)

where higher order corrections are discussed in the Supplementary Materials. Thus, for sufficiently large *V*/*t*, the boson bandwidth first increases with Δ and then decreases when Δ becomes comparable to *V*. This opens up the possibility of using the displacement field as a tuning knob to crossover between different physical regimes.

### Superconductivity and phase separation

The formation of excitonic Cooper pairs from strong electron repulsion has important implications when our system is doped away from *v* = 1, as we address below. The large binding energy $E_{b}∼t$ gives rise to tightly bound pairs, which form charge-2*e* bosons moving on a triangular lattice of *A* sites with hopping −*t_b_*. Depending on doping and microscopic details, bosons on a triangular lattice exhibit different phases, including a superfluid phase where a boson condensate forms, phase separation, and a supersolid (*46*–*49*), which simultaneously develops a charge density wave and superfluidity.

In the following, we present further evidence that these phases can be accessed within our model by tuning Δ. Specifically, for small Δ and large *V*/*t*, we found in ED that excitonic Cooper pairs attract each other when placed on next-nearest-neighbor sites, as detailed in the Supplementary Materials . Therefore, at a small doping δ, our system (in which interaction is short ranged) exhibits phase separation, where doped particles segregate into one phase at a high density *v*′ > 1 + δ and the other phase is undoped *v* = 1 insulator. Our ED calculations of the ground state energy as a function of doping shows phase separation between *v* = 1 and *v*′ = 1 ± 1/3 in the infinite coupling limit *V* → ∞ and Δ = 0; see Supplementary Materials for details. Phase separation is frustrated by the inclusion of longer-range Coulomb repulsion (*50*). Experimentally, the range of electron-electron interaction can be tuned by varying the distance of the sample to metallic gates.

**Fig. 6.**
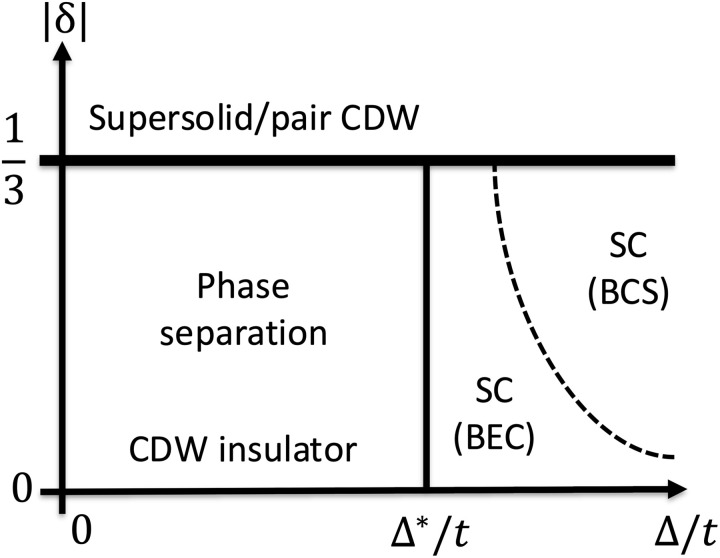
Schematic phase diagram as a function of doping |δ| and Δ/*t* at fixed *V*/*t*. For Δ < Δ*, the system undergoes phase separation between the charge density wave (CDW) insulator (low-density phase) and a pair CDW/supersolid state (high-density phase) with |δ| = 1/3. For Δ > Δ*, superconductivity (SC) emerges in the Bose-Einstein condensate (BEC) regime and undergoes a smooth crossover to the Bardeen–Cooper–Schrieffer (BCS) superconducting state at larger Δ/*t*.

On the other hand, upon increasing Δ at fixed large *V*/*t*, we find from ED calculations (see Supplementary Materials) that the interaction between excitonic Cooper pairs changes from attractive to repulsive above a critical value Δ*. For *V*/*t* = 8, our ED calculation shows $\Delta^{*}/t∼1$. At Δ > Δ*, our system with δ doped particles behaves as a two-dimensional Bose gas with repulsive interaction. Therefore, the ground state is a superfluid where charge-2*e* bosons condense in the q=0 state, leading to spin-polarized superconductivity from the condensation of excitonic Cooper pairs. The various competing phases are summarized in Fig. 6.

At small doping, the superfluid density is small despite the large binding energy *E*_b_. Thus, the superconducting critical temperature is governed by phase ordering (*51*–*55*). To estimate *T*_c_ of superconductivity of a gas of excitonic Cooper pairs, we use the expression (*56*–*61*)

$$k_{B}T_{c}\approx C\frac{\hbar^{2}\rho }{m_{b}}=C\frac{W}{3\sqrt{3}}\left|\delta \right|$$ (11)

where ρ=∣δ∣/(2Ω) is the density of pairs with $\Omega =\sqrt{3}a^{2}/2$ unit cell area and *W* is the bandwidth. In Eq. 11, *C* depends very weakly on the repulsive interaction between bosons through a double log (*56*–*58*, *62*); we set C≈2π/log(380/4π).

*T*_c_ (11) depends linearly on the doping density |δ|, in contrast to weak-coupling results where $T_{c}\propto \sqrt{\epsilon_{F}}$ (*9*) and the Fermi energy $\epsilon_{F}$ is proportional to |δ|. We note that, in the absence of gate screening (i.e., for 1/*r* Coulomb interaction), the *T* = 0 ground state of charged bosons at very low density is a Wigner crystal, whereas the superconducting state occurs above a critical density *r*_s_ < 60 (*63*). At temperatures above *T*_c_ and below the binding energy *E*_b_, we have a pseudogap regime where incoherent excitonic Cooper pairs constitute the charge carriers (*64*–*66*).

The increase of *T*_c_ with doping (11) breaks down when the average distance between excitonic Cooper pairs shrinks to its size. This sets an upper bound on *T*_c_, realized at boson density $\rho =1/\left(\pi \langle r^{2}\rangle \right)$ with $\langle r^{2}\rangle$ pair’s mean square radius that corresponds to the filling factor $|\delta |=\sqrt{3}a^{2}/\left(\pi \langle r^{2}\rangle \right)\approx 0.55a^{2}/\langle r^{2}\rangle$. For *V*/*t* = 8 and Δ/ *t* > 1 (where bosons repel), our ED calculations show that $\langle r^{2}\rangle \approx 1.25a^{2}$, leading to a critical temperature $k_{B}T_{c}\approx 0.06t$. For realistic parameters *t* = 2.5 meV and ϵ ≈ 7.5, this results in *T*_c_ = 1.7 K. Such parameter values lie within the range estimated from Wannierization of Γ-valley twisted TMD systems, indicating that the strong-coupling regime required for this estimate is experimentally feasible.

## DISCUSSION

Among various mechanisms for superconductivity from repulsive interaction, the most widely studied is pairing due to spin fluctuation, especially near magnetic quantum critical points. Our work presents a diametrically opposite route to unconventional superconductivity. For fully spin-polarized systems, which are completely devoid of spin fluctuation, we show that electron pairing can arise upon doping from particle-hole fluctuations in a correlated insulator, and the underlying Cooper pair is strongly hybridized with the exciton.

In contrast to previous approaches that integrate out the filled sublattice or treat excitonic fluctuations only perturbatively, our framework retains the full quantum dynamics of charge fluctuations and captures a strongly bound excitonic regime already at Δ < *t*. This unified treatment provides access to the strong coupling superconducting regime, the phase-separated region, the high-density *v* = 2/3 and *v* = 4/3 phases, and the quantum critical point at *V*_c_ and Δ = 0.

Twisted Γ-valley TMDs are a promising platform for realizing our honeycomb lattice model. In this setting, strong Coulomb repulsion induces sublattice polarization (*24*) and further drives ferromagnetism, as shown by our ED study of Γ-valley *t*TMDs and see also (*29*–*31*). This, in turn, establishes the parent state from which excitonic Cooper pair and superconductivity may emerge at finite doping. Experimental evidence for excitonic Cooper pairs can be obtained from electrostatic probes of the charged excitation spectrum of the parent insulating phase, with signatures expected to persist even without global phase coherence in the strong-coupling regime. Complementary, indirect evidence for our mechanism includes signatures of the unconventional *f*-wave symmetry of the pairs, accessible via phase-sensitive Josephson experiments and, upon doping, through the emergence of nodal Bogoliubov quasiparticles that affect thermodynamic observables; the appearance of a pseudogap regime above *T*_c_; and a tendency toward phase separation at low doping and small applied displacement fields when the interaction is sufficiently screened. 

Ferromagnetic superconductivity has been observed in rhombohedral graphene within both the spin-polarized, valley-unpolarized half-metal phase (*67*, *68*) and the valley-polarized quarter-metal phase (*69*). It will be interesting to explore the possibility of electron-hole fluctuations as a pairing mechanism, which can mediate intervalley pairing in the half-metal state corresponding to q=0 and give rise to *f*-wave superconductivity consistent with the pairing symmetry identified in our theory. In the quarter-metal phase, fluctuations associated with proximity to a charge-density-wave instability can favor pairing, although the symmetry of the resulting pairing remains to be determined.

## MATERIALS AND METHODS

ED calculations were performed on finite-size clusters in a torus geometry with periodic boundary conditions. The low-energy effective Hamiltonian was derived using a Schrieffer-Wolff transformation up to second order in *t*/*V*, starting from the *V* → ∞ strong-coupling limit and allowing for arbitrary values of Δ/*t*. Details of the projection onto the low-energy manifold, finite-size scaling analysis, and numerical implementation are provided in the Supplementary Materials.

## References

[1] W. Kohn, J. M. Luttinger, New mechanism for superconductivity. Phys. Rev. Lett. 15, 524–526 (1965).

[2] A. V. Chubukov, Kohn-Luttinger effect and the instability of a two-dimensional repulsive fermi liquid at t=0. Phys. Rev. B 48, 1097–1104 (1993). 10.1103/physrevb.48.1097 10007968

[3] J. González, Kohn-Luttinger superconductivity in graphene. Phys. Rev. B 78, 205431 (2008).

[4] S. Raghu, S. A. Kivelson, D. J. Scalapino, Superconductivity in the repulsive hubbard model: An asymptotically exact weak-coupling solution. Phys. Rev. B 81, 224505 (2010).

[5] A. Ghazaryan, T. Holder, M. Serbyn, E. Berg, Unconventional superconductivity in systems with annular fermi surfaces: Application to rhombohedral trilayer graphene. Phys. Rev. Lett. 127, 247001 (2021). 34951779 10.1103/PhysRevLett.127.247001

[6] T. Cea, P. A. Pantaleón, V. o. T. Phong, F. Guinea, Superconductivity from repulsive interactions in rhombohedral trilayer graphene: A Kohn-Luttinger-like mechanism. Phys. Rev. B 105, 075432 (2022).

[7] C. Schrade, L. Fu, Nematic, chiral, and topological superconductivity in twisted transition metal dichalcogenides. Phys. Rev. B 110, 035143 (2024).

[8] K. Slagle, L. Fu, Charge transfer excitations, pair density waves, and superconductivity in moiré materials. Phys. Rev. B 102, 235423 (2020).

[9] V. Crépel, L. Fu, New mechanism and exact theory of superconductivity from strong repulsive interaction. Sci. Adv. 7, eabh2233 (2021). 34301605 10.1126/sciadv.abh2233 PMC8302135

[10] V. Crépel, L. Fu, Spin-triplet superconductivity from excitonic effect in doped insulators. Proc. Natl. Acad. Sci. U.S.A. 119, e2117735119 (2022). 35320044 10.1073/pnas.2117735119 PMC9060479

[11] V. Crépel, T. Cea, L. Fu, F. Guinea, Unconventional superconductivity due to interband polarization. Phys. Rev. B 105, 094506 (2022).

[12] Y.-Z. Chou, F. Wu, S. Das Sarma, Enhanced superconductivity through virtual tunneling in bernal bilayer graphene coupled to wse_2_. Phys. Rev. B 106, L180502 (2022).

[13] Y. He, K. Yang, J. B. Profe, E. J. Bergholtz, D. M. Kennes, Superconductivity of repulsive spinless fermions with sublattice potentials. Phys. Rev. Res. 5, L012009 (2023).

[14] L. Homeier, H. Lange, E. Demler, A. Bohrdt, F. Grusdt, Feshbach hypothesis of high-tc superconductivity in cuprates. arXiv:2312.02982 [cond-mat.str-el] (2023). 10.1038/s41467-024-55549-4 PMC11696692 39747881

[15] V. Crépel, D. Guerci, J. Cano, J. H. Pixley, A. Millis, Topological superconductivity in doped magnetic moiré semiconductors. Phys. Rev. Lett. 131, 056001 (2023). 37595206 10.1103/PhysRevLett.131.056001

[16] H. Yang, H. Oh, Y.-H. Zhang, Strong pairing from a small fermi surface beyond weak coupling: Application to la_3_ni_2_o_7_. Phys. Rev. B 110, 104517 (2024).

[17] J. von Milczewski, X. Chen, A. Imamoglu, R. Schmidt, Superconductivity induced by strong electron-exciton coupling in doped atomically thin semiconductor heterostructures. Phys. Rev. Lett. 133, 226903 (2024). 39672128 10.1103/PhysRevLett.133.226903

[18] C. Zerba, C. Kuhlenkamp, A. Imamoğlu, M. Knap, Realizing topological superconductivity in tunable bose-fermi mixtures with transition metal dichalcogenide heterostructures. Phys. Rev. Lett. 133, 056902 (2024). 39159121 10.1103/PhysRevLett.133.056902

[19] Y. Takahashi, H. Miyamoto, K. Kuroki, T. Kaneko, Floquet engineering of effective pairing interactions in a doped band insulator. Phys. Rev. B 111, 125104 (2025).

[20] Y. Zhang, T. Liu, L. Fu, Electronic structures, charge transfer, and charge order in twisted transition metal dichalcogenide bilayers. Phys. Rev. B 103, 155142 (2021).

[21] M. Angeli, A. H. MacDonald, Γ valley transition metal dichalcogenide moiré bands. Proc. Natl. Acad. Sci. U.S.A. 118, e2021826118 (2021). 33658375 10.1073/pnas.2021826118 PMC7958387

[22] L. Xian, M. Claassen, D. Kiese, M. M. Scherer, S. Trebst, D. M. Kennes, A. Rubio, Realization of nearly dispersionless bands with strong orbital anisotropy from destructive interference in twisted bilayer MoS_2_. Nat. Commun. 12, 5644 (2021). 34561454 10.1038/s41467-021-25922-8 PMC8463715

[23] H. Pan, E.-A. Kim, C.-M. Jian, Realizing a tunable honeycomb lattice in abba-stacked twisted double bilayer WSe_2_. Phys. Rev. Res. 5, 043173 (2023).

[24] L. Ma, R. Chaturvedi, P. X. Nguyen, K. Watanabe, T. Taniguchi, K. F. Mak, J. Shan, Relativistic mott transition in strongly correlated artificial graphene. arXiv:2412.07150 [cond-mat.mes-hall] (2024).

[25] D. M. Kennes, M. Claassen, L. Xian, A. Georges, A. J. Millis, J. Hone, C. R. Dean, D. N. Basov, A. N. Pasupathy, A. Rubio, Moiré heterostructures as a condensed-matter quantum simulator. Nat. Phys. 17, 155–163 (2021).

[26] K. F. Mak, J. Shan, Semiconductor moiré materials. Nat. Nanotechnol. 17, 686–695 (2022). 35836003 10.1038/s41565-022-01165-6

[27] Y. Wang, J. Choe, E. Anderson, W. Li, J. Ingham, E. A. Arsenault, Y. Li, X. Hu, T. Taniguchi, K. Watanabe, X. Roy, D. Basov, D. Xiao, R. Queiroz, J. C. Hone, X. Xu, X. Y. Zhu, Hidden states and dynamics of fractional fillings in twisted MoTe_2_ bilayers. Nature 641, 1149–1155 (2025). 40179960 10.1038/s41586-025-08954-8

[28] A. Kormányos, G. Burkard, M. Gmitra, J. Fabian, V. Zólyomi, N. D. Drummond, V. Fal’ko, k⸱p Theory for two-dimensional transition metal dichalcogenide semiconductors. 2D Materials 2, 022001 (2015).

[29] Y. Yang, M. A. Morales, S. Zhang, Ferromagnetic semimetal and charge-density wave phases of interacting electrons in a honeycomb moiré potential. Phys. Rev. Lett. 133, 266501 (2024). 39878996 10.1103/PhysRevLett.133.266501

[30] Y. Zhang, Y. Zhang, Insulating charge transfer ferromagnetism. arXiv:2410.22399 [cond-mat.str-el] (2024).

[31] T. Devakul, V. Crépel, Y. Zhang, L. Fu, Magic in twisted transition metal dichalcogenide bilayers. Nat. Commun. 12, 6730 (2021). 34795273 10.1038/s41467-021-27042-9 PMC8602625

[32] Y. Nagaoka, Ferromagnetism in a narrow, almost half-filled *s* band. Phys. Rev. 147, 392–405 (1966).

[33] D. J. Gross, A. Neveu, Dynamical symmetry breaking in asymptotically free field theories. Phys. Rev. D 10, 3235–3253 (1974).

[34] G. W. Semenoff, Condensed-matter simulation of a three-dimensional anomaly. Phys. Rev. Lett. 53, 2449–2452 (1984).

[35] I. F. Herbut, V. Juričić, O. Vafek, Relativistic mott criticality in graphene. Phys. Rev. B 80, 075432 (2009).

[36] I. F. Herbut, Interactions and phase transitions on graphene’s honeycomb lattice. Phys. Rev. Lett. 97, 146401 (2006). 17155272 10.1103/PhysRevLett.97.146401

[37] V. Juričić, I. F. Herbut, G. W. Semenoff, Coulomb interaction at the metal-insulator critical point in graphene. Phys. Rev. B 80, 081405 (2009).

[38] L. Wang, P. Corboz, M. Troyer, Fermionic quantum critical point of spinless fermions on a honeycomb lattice. New J. Phys. 16, 103008 (2014).

[39] L. Wang, Y.-H. Liu, M. Troyer, Stochastic series expansion simulation of the *t*−*v* model. Phys. Rev. B 93, 155117 (2016).

[40] S. Capponi, Phase diagram of interacting spinless fermions on the honeycomb lattice. J. Phys. Condens. Matter 29, 043002 (2017). 27875325 10.1088/1361-648X/29/4/043002

[41] E. Huffman, S. Chandrasekharan, Fermion bag approach to Hamiltonian lattice field theories in continuous time. Phys. Rev. D 96, 114502 (2017).

[42] M. Schuler, S. Hesselmann, S. Whitsitt, T. C. Lang, S. Wessel, A. M. Läuchli, Torus spectroscopy of the gross-neveu-yukawa quantum field theory: Free dirac versus chiral ising fixed point. Phys. Rev. B 103, 125128 (2021).

[43] J. R. Schrieffer, P. A. Wolff, Relation between the Anderson and Kondo Hamiltonians. Phys. Rev. 149, 491–492 (1966).

[44] A. H. MacDonald, S. M. Girvin, D. Yoshioka, $\frac{t}{U}$ expansion for the hubbard model. Phys. Rev. B 37, 9753 (1988). 10.1103/physrevb.37.9753 9944372

[45] S. Bravyi, D. P. DiVincenzo, D. Loss, Schrieffer–wolff transformation for quantum many-body systems. Ann. Phys. Rehabil. Med. 326, 2793–2826 (2011).

[46] S. Wessel, M. Troyer, Supersolid hard-core bosons on the triangular lattice. Phys. Rev. Lett. 95, 127205 (2005). 16197105 10.1103/PhysRevLett.95.127205

[47] R. G. Melko, A. Paramekanti, A. A. Burkov, A. Vishwanath, D. N. Sheng, L. Balents, Supersolid order from disorder: Hard-core bosons on the triangular lattice. Phys. Rev. Lett. 95, 127207 (2005). 16197107 10.1103/PhysRevLett.95.127207

[48] G. Murthy, D. Arovas, A. Auerbach, Superfluids and supersolids on frustrated two-dimensional lattices. Phys. Rev. B 55, 3104–3121 (1997).

[49] K. Bernardet, G. G. Batrouni, J.-L. Meunier, G. Schmid, M. Troyer, A. Dorneich, Analytical and numerical study of hardcore bosons in two dimensions. Phys. Rev. B 65, 104519 (2002).

[50] R. Jamei, S. Kivelson, B. Spivak, Universal aspects of coulomb-frustrated phase separation. Phys. Rev. Lett. 94, 056805 (2005). 15783677 10.1103/PhysRevLett.94.056805

[51] J. M. Kosterlitz, D. J. Thouless, Ordering, metastability and phase transitions in two-dimensional systems. J. Phys. C. Solid State Phys. 6, 1181–1203 (1973). 10.1088/0953-8984/28/48/481001 27665689

[52] D. R. Nelson, J. M. Kosterlitz, Universal jump in the superfluid density of two-dimensional superfluids. Phys. Rev. Lett. 39, 1201–1205 (1977).

[53] M. Randeria, N. Trivedi, A. Moreo, R. T. Scalettar, Pairing and spin gap in the normal state of short coherence length superconductors. Phys. Rev. Lett. 69, 2001–2004 (1992). 10046371 10.1103/PhysRevLett.69.2001

[54] V. Emery, S. Kivelson, Importance of phase fluctuations in superconductors with small superfluid density. Nature 374, 434–437 (1995).

[55] T. Hazra, N. Verma, M. Randeria, Bounds on the superconducting transition temperature: Applications to twisted bilayer graphene and cold atoms. Phys. Rev. X 9, 031049 (2019).

[56] D. S. Fisher, P. C. Hohenberg, Dilute bose gas in two dimensions. Phys. Rev. B 37, 4936–4943 (1988). 10.1103/physrevb.37.4936 9943665

[57] N. Prokof'ev, O. Ruebenacker, B. Svistunov, Critical point of a weakly interacting two-dimensional bose gas. Phys. Rev. Lett. 87, 270402 (2001). 11800861 10.1103/PhysRevLett.87.270402

[58] S. Pilati, S. Giorgini, N. Prokof'ev, Critical temperature of interacting bose gases in two and three dimensions. Phys. Rev. Lett. 100, 140405 (2008). 18518010 10.1103/PhysRevLett.100.140405

[59] C. Zhang, B. Capogrosso-Sansone, M. Boninsegni, N. V. Prokof'ev, B. V. Svistunov, Superconducting transition temperature of the bose one-component plasma. Phys. Rev. Lett. 130, 236001 (2023). 37354424 10.1103/PhysRevLett.130.236001

[60] C. Zhang, J. Sous, D. R. Reichman, M. Berciu, A. J. Millis, N. V. Prokof'ev, B. V. Svistunov, Bipolaronic high-temperature superconductivity. Phys. Rev. 13, 011010 (2023).

[61] K.-S. Kim, Z. Han, J. Sous, Semiclassical theory of bipolaronic superconductivity in a bond-modulated electron-phonon model. Phys. Rev. B 109, L220502 (2024).

[62] V. N. Popov, On the theory of the superfluidity of two- and one-dimensional bose systems. Theor. Math. Phys. 11, 565–573 (1972).

[63] S. De Palo, S. Conti, S. Moroni, Monte carlo simulations of two-dimensional charged bosons. Phys. Rev. B 69, 035109 (2004).

[64] D. M. Eagles, Possible pairing without superconductivity at low carrier concentrations in bulk and thin-film superconducting semiconductors. Phys. Rev. 186, 456–463 (1969).

[65] A. J. Leggett, “Diatomic molecules and cooper pairs,” in *Modern Trends in the Theory of Condensed Matter*, A. Pekalski, J. A. Przystawa, Eds. (Springer, 1980), pp. 13–27.

[66] M. Randeria, E. Taylor, Crossover from bardeen-cooper-schrieffer to bose-einstein condensation and the unitary fermi gas. Annu. Rev. Condens. Matter Phys. 5, 209–232 (2014).

[67] H. Zhou, T. Xie, T. Taniguchi, K. Watanabe, A. F. Young, Superconductivity in rhombohedral trilayer graphene. Nature 598, 434–438 (2021). 34469942 10.1038/s41586-021-03926-0

[68] H. Zhou, L. Holleis, Y. Saito, L. Cohen, W. Huynh, C. L. Patterson, F. Yang, T. Taniguchi, K. Watanabe, A. F. Young, Isospin magnetism and spin-polarized superconductivity in bernal bilayer graphene. Science 375, 774–778 (2022). 35025604 10.1126/science.abm8386

[69] T. Han, Z. Lu, Z. Hadjri, L. Shi, Z. Wu, W. Xu, Y. Yao, A. A. Cotten, O. S. Sedeh, H. Weldeyesus, J. Yang, J. Seo, S. Ye, M. Zhou, H. Liu, G. Shi, Z. Hua, K. Watanabe, T. Taniguchi, P. Xiong, D. M. Zumbühl, L. Fu, L. Ju, Signatures of chiral superconductivity in rhombohedral graphene. arXiv:2408.15233 [cond-mat.mes-hall] (2025). 10.1038/s41586-025-09169-7 40403766

