# Supplementary material for: Ferromagnetic superconductivity with excitonic Cooper pairs: Application to Γ-valley twisted semiconductors
Source: Sci Adv. 2026 May 20;12(21):eaeb4888. doi: 10.1126/sciadv.aeb4888 (PMC13189127; doi:10.1126/sciadv.aeb4888)
Supplement: Supplementary file 1 — Supplementary Text Figs. S1 to S28 [file sciadv.aeb4888_sm.pdf]

Science Advances
AAAS

Supplementary Materials for

Ferromagnetic superconductivity with excitonic Cooper pairs: Application to Γ-valley twisted semiconductors

Daniele Guerci and Liang Fu

Corresponding author: Daniele Guerci, danieleguerci@hotmail.it; Liang Fu, liangfu@mit.edu

Sci. Adv. **12**, eaeb4888 (2026)
DOI: 10.1126/sciadv.aeb4888

This PDF file includes:

Supplementary Text
Figs. S1 to S28

These supplementary materials contain the details of the continuum and tight-binding modeling of Γ-valley moiré semiconductors, exact diagonalization simulations and strong coupling perturbative results supporting our theory of exciton Cooper pairing in half metals. Sec. A provides a detailed discussion of the interacting properties of twisted Γ-valley semiconductors, including the continuum model, interaction effects, the resulting many-body physics and the tight-binding modeling. In Sec. B we present details on exact diagonalization simulations of the tight-binding model. Sec. C focuses on $V \to \infty$ strong coupling limit, presenting exact analytical results for one and two doped carriers, along with numerical studies of many-body physics involving N doped carriers in the spin charge density wave ground state. Sec. D presents the analytical $1/V$ perturbation theory providing variational estimates to the boson dispersion relation, binding energy and ground state energy. Finally, analytical results valid in the ionic regime $\Delta \gg t$ are given in Sec. E.

Appendix A: Microscopic modeling of Γ-valley twisted semiconductors

Γ-valley moiré semiconductors are described by the continuum model introduced in Ref. [1]:

$$H(r) = -\frac{k^2}{2m} + \begin{pmatrix} u_t(r) + D/2 & t(r) \\ t(r) & u_b(r) - D/2 \end{pmatrix}, \tag{S1}$$

where in the previous expression $k = -i\hbar\nabla$, $u_{t/b}(r) = 2V_0 \sum_{j=1}^{3} \cos(g_j \cdot r \pm \phi)$, $t(r) = w_0 + 2w_1 \sum_{j=1}^{3} \cos(g_j \cdot r) + 2w_2 \sum_{j=1}^{3} \cos(2g_j \cdot r)$ and D is the displacement field. We fix the reference frame such that $a_j = a \exp[i\pi/2 + 2i\pi(j-1)/3]$ and $g_j = 4\pi\omega^{j-1}/(\sqrt{3}a)$ with $\omega = \exp(2\pi i/3)$ and $a = a_0/(2\sin\theta/2)$ with a_0 the atomic lattice constant, where we utilized complex notation.

We employed the parameters $w_0 = 338$meV, $w_1 = -16$meV, $w_2 = -2$meV, $V_0 = 6$meV and $\phi = 120°$ derived for MoS_2 in Ref. [1], and the effective mass $m = 0.8m_e$. Fig. S3 displays the bandstructure for different values of the displacement field D.

The large interlayer energy scale w_0 implies that the topmost bands are predominantly characterized by a layer-bonding configuration with only small layer imbalance $\gamma^z = \mathrm{diag}[1, -1]$. As a result, the influence of the displacement field on the band structure is small when it is less than the interlayer bonding energy w_0, e.g. for a twist angle $\theta = 2.876°$ the sublattice gap is 1meV for $D = 30$meV and 3.5meV for $D = 100$meV.

The model is invariant under the three-fold rotational symmetry C_{3z}, two-fold rotations C_{2y}, the three-dimensional inversion $\gamma^x H(-r)\gamma^x = H(r)$ with γ^x Pauli matrix in the layer degree of freedom and M_y mirror symmetry $y \to -y$. Moreover, the model preserves time-reversal symmetry ($\mathcal{T}$) and SU(2) spin rotational symmetry, as the spin-orbit coupling is negligible at Γ [2]. We note that C_{2y} and the three-dimensional inversion are broken either spontaneously (at filling $\nu = 1$) when $D = 0$ and above a critical interaction strength or explicitly when $D \neq 0$ in the sublattice-polarized insulator. Additionally, in the spin-polarized regime—whether induced by interactions [3, 4] or an applied in-plane magnetic field—both $\mathcal{T}$ and $SU(2)_{\mathrm{spin}}$ symmetry are broken.

1. Interacting continuum model and exact diagonalization results

The many-body Hamiltonian reads:

$$H = \sum_i H(r_i) + \frac{1}{2}\sum_{i\neq j} V(r_i - r_j), \tag{S2}$$

where $H(r_i)$ is the single-particle Hamiltonian given in Eq. (S1). We considered the double-gate screened Coulomb interaction, which in momentum space reads:

$$V(q) = \frac{e^2}{2\epsilon_0\epsilon}\frac{1}{q}\tanh d_{\mathrm{sc}}q, \tag{S3}$$

where d_{sc} is the gate distance and ϵ the relative dielectric constant. The interacting physics is characterized by the competition of two energy scales: the kinetic energy $E_{\mathrm{k}} = \hbar^2/(2ma^2)$ and the interaction energy $E_{\mathrm{int}} = e^2 a/(2\epsilon_0\epsilon|a_1 \times a_2|)$. Tendency to ferromagnetism is enhanced in the small twist angle regime, where the bandwidth is much smaller than the interaction energy scale. We perform exact diagonalization simulations projecting the Hamiltonian in the two topmost bands with dispersion E_{kn}:

$$H = \sum_k \sum_n \sum_\sigma E_{kn} c^\dagger_{kn\sigma} c_{kn\sigma} + \frac{1}{2A}\sum_{\sigma_1\cdots\sigma_4}\sum_{n_1\cdots n_4}\sum_{k_1\cdots k_4} H_{q_1q_2,q_3q_4} c^\dagger_{k_1n_1\sigma_1} c^\dagger_{k_2n_2\sigma_2} c_{k_3n_3\sigma_3} c_{k_4n_4\sigma_4}, \tag{S4}$$

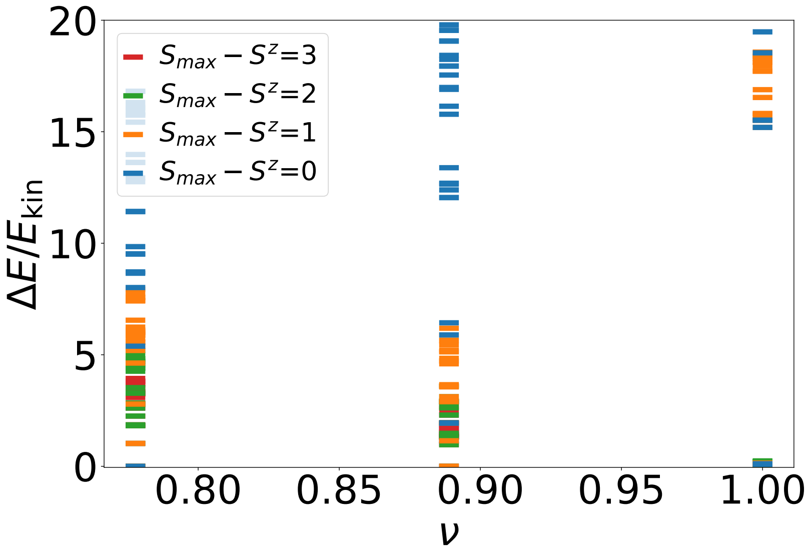

FIG. S1. Many-body spectrum as a function of the filling factor ν. The ground state sector features high-spin configurations evidence of tendency to form a ferromagnetic state. Calculations are performed setting $\theta = 2^\circ$, $d_{\rm sc} = 5$nm and $\epsilon = 10$.

where $c_{kn\sigma}$ is the annihilation operator for an electron with momentum k, band index n and spin σ, and $A = |L_1 \times L_2|$. Furthermore, we have introduced the label $q_j = (k_j, n_j, \sigma_j)$ in the interaction matrix element. For a given set of indices the resulting matrix element reads

$$H_{q_1 q_2, q_3 q_4} = \delta_{\sigma_1 \sigma_4} \delta_{\sigma_2 \sigma_3} \sum_g \delta_{k_1+k_2-k_3-k_4,\Delta g} \, V(k_1 - k_4 - g) \, \Lambda^{n_1,n_4}_{k_1,k_4+g} \, \Lambda^{n_2,n_3}_{k_2,k_3+\Delta g - g}, \tag{S5}$$

where we have introduced:

$$\Lambda^{n,m}_{k,p+g} = \int_{\rm UC} \frac{d^2 r}{\Omega} e^{-ig\cdot r} u^*_{kn}(r) u_{pm}(r), \tag{S6}$$

where $\Omega = |a_1 \times a_2|$, $|u_{k\pm}\rangle$ are the Bloch waves associated with the two low-energy bands hosting the Dirac cone.

We perform exact diagonalization simulations on a 3×3 cluster that includes the two low-energy bands and both spin degrees of freedom. Fig. S1 shows the many-body spectrum for filling factors $\nu = 7/9$, $8/9$, and 1, all exhibiting extensive ground-state degeneracy, reflected in the high-spin configurations that characterize the ground states. Interestingly, while the states at $\nu = 1$ and $7/9$ exhibit the full $(2S+1)$ spin degeneracy, the $\nu = 8/9$ state—corresponding to a single doped hole—displays only a $(2S-1)$ degeneracy. This reduction implies that the added hole forms a spin-singlet bound state, signaling the onset of polaronic physics. Furthermore, we observe that at filling factor $\nu = 1$, the spin-polarized ground state sector is two-fold degenerate, with each ground state spontaneously breaking the C_{2y} symmetry by localizing the charge distribution on one of the two sublattices as detailed in Fig. S2.

2. Limit of a strong moiré potential and sublattice basis

In this section we take the limit of large moiré potential and we expand around the minima to determine the localization length of the orbitals. Our goal is to construct the basis that will be used to define the Wannier orbitals through projection [5, 6]. The orbitals are localized around Wyckoff positions $\pm z_0 = \pm(a_1 - a_2)/3$ with $a_{1/2} = e^{-i\pi/6}, e^{i\pi/2}$. Expanding around these points we find:

$$u_b(z_0 + \delta r) \approx 6V_0 - \frac{4\pi^2 (2V_0)}{a^2} \delta r^2, \quad u_t(z_0 + \delta r) \approx -3V_0 + \frac{4\pi^2 V_0}{a^2} \delta r^2, \tag{S7}$$

in addition we have the expansion of the potential $\Delta(r)$:

$$\Delta(z_0 + \delta r) \approx \bar{w} + \frac{4\pi^2}{a^2} (w_1 + 4w_2) \, \delta r^2, \tag{S8}$$

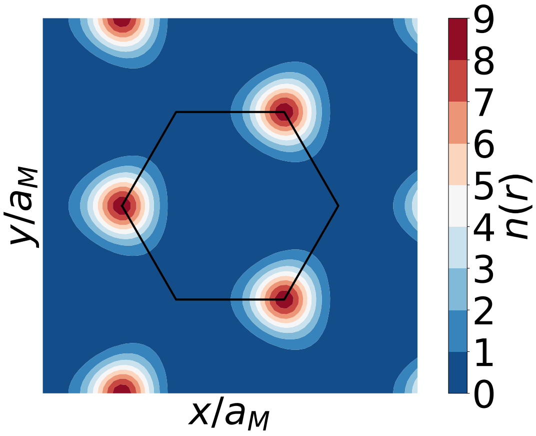

FIG. S2. Charge distribution $n(r) = \langle\Psi|\psi^\dagger(r)\psi(r)|\Psi\rangle$ with $|\Psi\rangle$ the many-body ground state. Calculations are performed setting $\theta = 2^\circ$, $d_{\rm sc} = 5$nm, $\epsilon = 10$ and a small displacement field D ($D = .05$meV) applied to lift the degeneracy between the two ground states polarized on opposite sublattices.

$\bar{w} = w_0 - 3(w_1 + w_2) = 392$meV and $\Delta w = w_1 + 4w_2 = -24$meV. We observe that the difference in $\epsilon_{b/t}$ introduces an asymmetry between $\pm z_0$ high symmetry stackings leading to a finite out-of-plane polarization of the orbitals resulting in a net response to an applied displacement field. Around z_0, the Hamiltonian is approximated as:

$$H(r + z_0) \approx \frac{9V_0}{2}\sigma^z + \bar{w}\sigma^x - \frac{k^2}{2m} + \frac{4\pi^2\delta r^2}{a^2}\begin{pmatrix} -2V_0 & \Delta w \\ \Delta w & V_0 \end{pmatrix}, \tag{S9}$$

where $9V_0/(2\bar{w}) \approx 0.07 \ll 1$ implying that the state is mostly described by a layer symmetric configuration. The eigenstates diagonalizing the potential are given by:

$$|v_+\rangle = [\cos\chi/2, \sin\chi/2]^T, \quad |v_-\rangle = [-\sin\chi/2, \cos\chi/2]^T, \tag{S10}$$

with $\chi = \arctan \Delta_x/\Delta_z$ and $\Delta_x = \bar{w}$, $\Delta_z = 9V_0/2 + D/2$ if a displacement field is applied. The energy gap between the two states $v_\pm$ is large and we perform projection to the topmost state $|v_+\rangle$. Notice that $\langle v_+|\boldsymbol{\tau}|v_+\rangle = (0.9976, 0, 0.0687)$ is remarkably close to the layer distribution obtained from the Bloch state at z_0: $\left\langle \boldsymbol{\psi}_{\gamma 1/2}(z_0) \middle| \boldsymbol{\tau} \middle| \boldsymbol{\psi}_{\gamma 1/2}(z_0) \right\rangle = (0.9980, 0, 0.0624), (0.9980, 0, 0.0635)$. We proceed simply projecting the space dependent part of the Hamiltonian in the topmost configuration obtaining:

$$\mathcal{H}_+ \equiv \langle v_+|H(r + z_0)|v_+\rangle = -\frac{k^2}{2m} - \frac{4\pi^2\delta r^2}{a^2}E_h, \quad E_h = \langle v_+|\begin{pmatrix} -2V_0 & \Delta w \\ \Delta w & V_0 \end{pmatrix}|v_+\rangle\,. \tag{S11}$$

The Hamiltonian in the hole like picture can be then written as:

$$\mathcal{H}_+ = \frac{\hbar^2 k^2}{2m} + \frac{\alpha r^2}{2a^2} \tag{S12}$$

with $\alpha = 8\pi^2 E_h$. We readily find the frequency of the harmonic oscillator ω, the localisation length ℓ and the wavefunction ψ_0:

$$\hbar\omega = \sqrt{\frac{8\pi^2\hbar^2}{ma^2}E_h}, \quad \frac{\ell}{a} = \left[\frac{\hbar^2}{8\pi^2 m a^2 E_h}\right]^{\frac{1}{4}}, \quad \psi_0(r) = \frac{e^{-r^2/(2\ell^2)}}{\ell\sqrt{\pi}}. \tag{S13}$$

The spread of the wavefunction decays with the square root of the twist angle as the twist angle is reduced as shown in Fig. S4. Therefore, we have two different low-energy localised states given by:

$$\boldsymbol{\psi}_+(r) = |v_{+,+}\rangle\,\frac{e^{-(r-z_0)^2/(2\ell^2)}}{\ell\sqrt{\pi}}, \quad \boldsymbol{\psi}_-(r) = |v_{+,-}\rangle\,\frac{e^{-(r-z_1)^2/(2\ell^2)}}{\ell\sqrt{\pi}}, \tag{S14}$$

where $z_1 = -\omega z_0$ and $|v_{+,\pm}\rangle$ the topmost energy eigenstates diagonalizing the moiré potential around $z_{0/1}$ (S9).

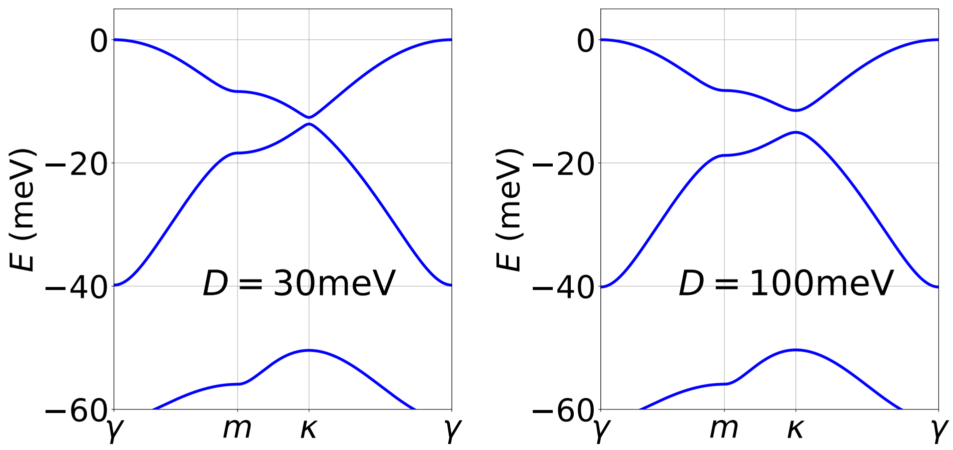

FIG. S3. Bandstructure of gamma valley moiré semiconductors for $D = 30, 100$meV. Applying the displacement field breaks C_{2y} and opens a trivial gap at κ and κ'. The twist angle is given by $\theta = 2.876°$.

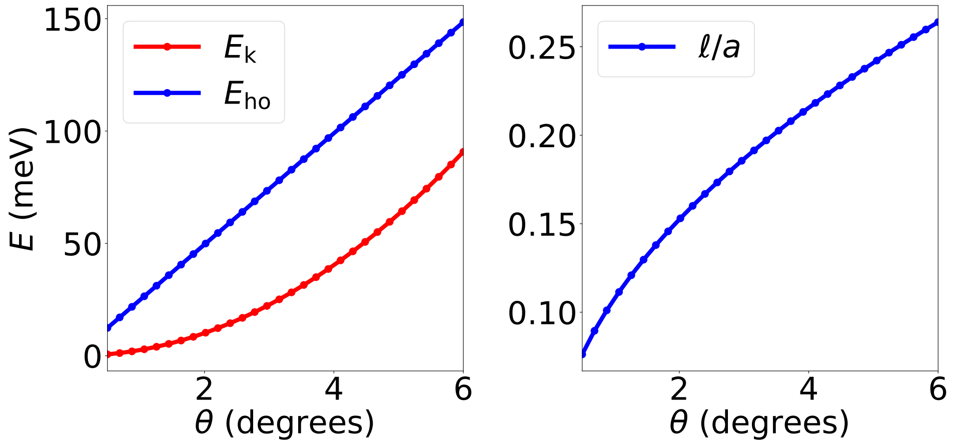

FIG. S4. Energy scaling for quadratic dispersion (red) and harmonic oscillator (blue). Localisation length of the lowest energy state in each well. Notice that the distance between the two quantum well is $a/\sqrt{3} \approx 0.58a$.

3. Wannier orbitals via projection

We now employ the states $\boldsymbol{\psi}_\pm$ (S14) to build the Wannier functions obtained of the two topmost bands. To this aim we start from the Bloch orbitals consisting of two component spinors $\boldsymbol{\psi}_k = [\psi_{kt}, \psi_{kb}]^T$ in the layer index $\ell = t, b$:

$$\boldsymbol{\psi}_{kn}(r) = e^{ik\cdot r} \sum_g \boldsymbol{z}_{ng}(k) e^{ig\cdot r}, \tag{S15}$$

where $g = ng_1 + mg_2$ with $n, m \in \mathbb{Z}$ are reciprocal lattice vectors and $\boldsymbol{z}_{ng}(k)$ a spinor in the layer degree of freedom. We employ the exact expressions for the wavefunctions in the large potential limit projecting the Bloch states into the set of states $\boldsymbol{\psi}_\pm(r) = |v_{+,\pm}\rangle f_\pm(r)$ where f is a Gaussian function centered at the potential minima (S14). To this aim we first introduce the projected basis:

$$\begin{aligned}\boldsymbol{\phi}_{kn}(r) &= \sum_m \boldsymbol{\psi}_{km}(r) \langle \psi_{km}|\psi_n\rangle = \sum_m \boldsymbol{\psi}_{km}(r) \sum_g \langle z_{mg}(k)|v_{+,n}\rangle \int d^2 r e^{-i(k+g)\cdot r} f_n(r) \\ &= \sum_m \boldsymbol{\psi}_{km}(r) \sum_g \langle z_{mg}(k)|v_{+,n}\rangle\, e^{-i(k+g)\cdot z_n} e^{-(k+g)^2 \ell_n^2/2}.\end{aligned} \tag{S16}$$

The new eigenstate basis $\boldsymbol{\phi}_{kn}$ serves as the starting point for constructing the Wannier function:

$$\tilde{\boldsymbol{\psi}}_{kn}(\boldsymbol{r}) = \sum_j \boldsymbol{\phi}_{kj}(r) S^{-1/2}_{jn}(k), \quad S(k) = A^\dagger(k) A(k), \tag{S17}$$

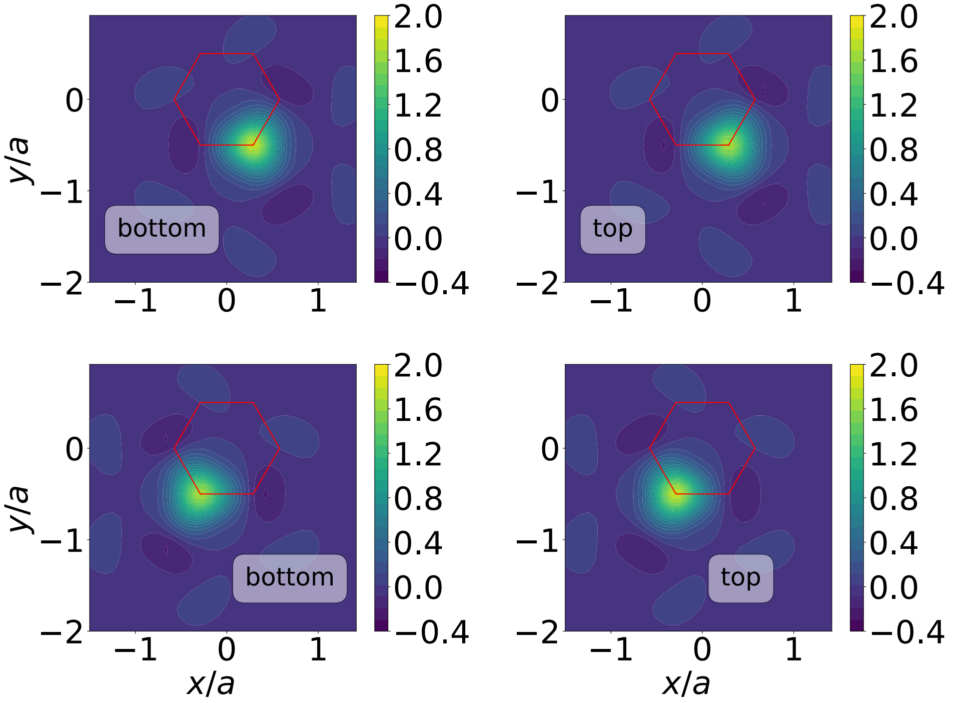

FIG. S5. Layer resolved Wannier functions for the two topmost bands of gamma valley twisted semiconductors for twist angle $\theta = 2.876°$ forming a honeycomb lattice. The left and right columns represent the bottom and top layers, respectively, while the upper and lower rows depict the Wannier functions $\boldsymbol{W}_{RA/B}(r)$ localized at the MX and XM high-symmetry stacking configurations, which form the two sublattices A/B of the emergent honeycomb lattice.

and we have introduced the matrix:

$$A_{mn}(k) = \sum_{\boldsymbol{g}} \langle z_{m\boldsymbol{g}}(k)|v_{+,n}\rangle \, e^{-i(k+\boldsymbol{g})\cdot z_n} e^{-(k+\boldsymbol{g})^2 \ell_n^2/2}. \tag{S18}$$

Notice that Eq. (S17) can be also expressed as:

$$\tilde{\boldsymbol{\psi}}_{kn}(\boldsymbol{r}) = \sum_j \boldsymbol{\psi}_{kj}(r) U_{jn}(k), \quad U(k) = A(k) S^{-1/2}(k) \tag{S19}$$

with $U(k)$ unitary matrix. Finally, the Wannier orbitals are given by:

$$\boldsymbol{W}_{Rn}(r) = \frac{1}{N}\sum_k \tilde{\boldsymbol{\psi}}_{kn}(r) e^{-ik\cdot R} = \frac{1}{N}\sum_k \tilde{\boldsymbol{\psi}}_{kn}(r) e^{-ik\cdot R}, \tag{S20}$$

with N number of unit cells and $R = na_1 + ma_2$ with $n, m \in \mathbb{Z}$.

The Wannier orbitals are shown in Fig. S5 for the twist angle $2.876°$ and $D = 0$. The Wannier orbitals show a slight layer imbalance ($\langle \boldsymbol{W}_{RB}|\gamma^z|\boldsymbol{W}_{RB}\rangle = -\langle \boldsymbol{W}_{RA}|\gamma^z|\boldsymbol{W}_{RA}\rangle \neq 0$) with $\gamma^z = \mathrm{diag}[1, -1]$ in the layer degree of freedom and transform into each other under C_{2y}.

4. Tunneling amplitudes, minimal lattice model and Coulomb repulsion

We now compute the hopping amplitudes:

$$t^{n,n'}_{R,R'} = \frac{1}{N}\sum_k \sum_{l=0,1} e^{ik\cdot(R-R')} U^\dagger_{nl}(k) E_{kl} U_{ln'}(k), \tag{S21}$$

where l extends only to the topmost twofold manifold of bands and $U(k)$ is the unitary transformation to the sublattice basis given in Eq. (S19).

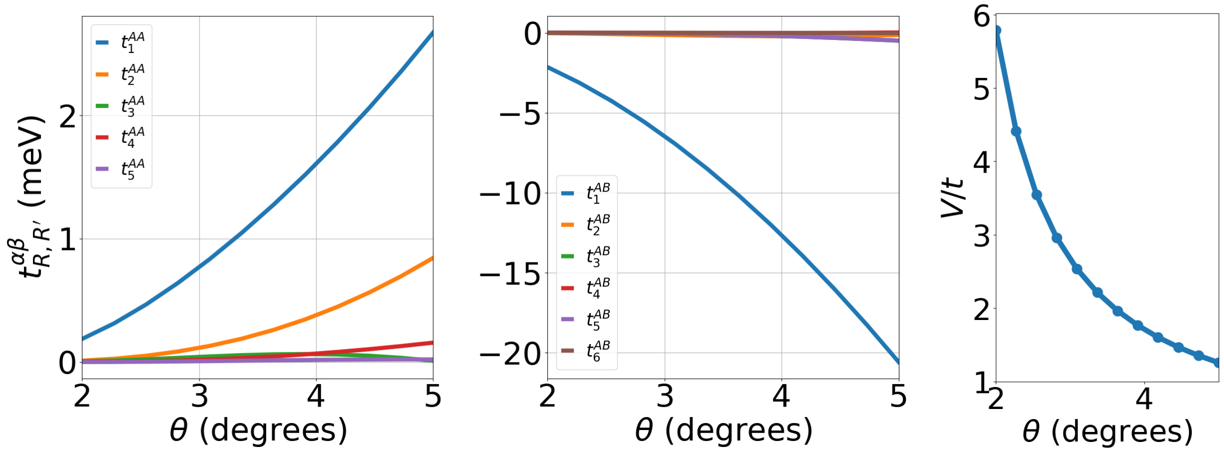

FIG. S6. Left panels show Tunneling amplitudes for vanishing displacement field $D = 0$. The right most panel shows the ratio V/t with V and t nearest-neighbor interaction and hopping, respectively. In this case, $\Delta = 0$ and $t_n^{AA} = t_n^{BB}$.

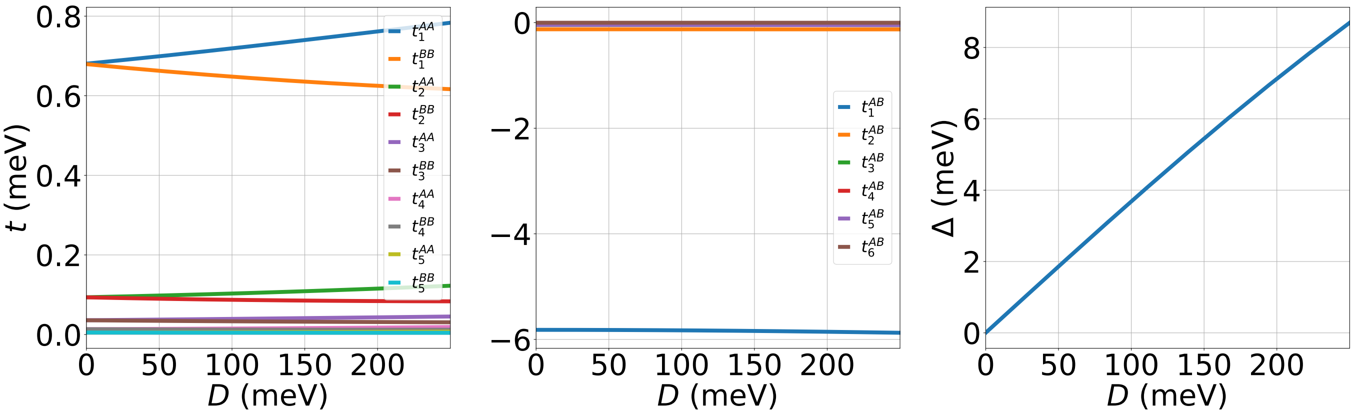

FIG. S7. Evolution of tight binding parameters with the displacement field $D = 0$ for $\theta = 2.876°$. The leading hopping process is the n.n. tunneling connecting the two sublattice. The right panel shows the evolution of Δ as a function of D. Due to the large interlayer energy scale w_0 given below Eq. (S1), the wavefunctions exhibit only a slight interlayer imbalance.

The evolution of the leading hopping terms $t_n^{AB} \equiv t_{a_n,0}^{AB}$, $t_n^{BB} = t_{a_n,0}^{BB}$, and $t_n^{AA} = t_{a_n,0}^{AA}$ as a function of the twist angle is given in Fig. S6 with subscript denoting the increasing number of shell in real space. For the intrasublattice hopping t^{AA}, t^{BB} the contribution of the higher shell decreases with increasing distance on the lattice, while the intersublattice hopping is dominated by the n.n. contribution. Furthermore, Fig. S7 shows the evolution of the hopping and sublattice gap as a function of the applied displacement field. The hopping t_2^A and t_2^B change differently for the two different sublattice degrees of freedom by increasing the displacement field D. Specifically, the state with smaller zero point energy is less confined, has a larger localization length and, therefore, a larger hopping amplitude. In momentum space the lattice model describing twisted Γ twisted homobilayers is in the basis $\Psi_k = [a_k, b_k]$:

$$H_k = \begin{pmatrix} \epsilon_{kA} + \Delta/2 & t_k \\ c.c. & \epsilon_{kB} - \Delta/2, \end{pmatrix} \tag{S22}$$

with a_j lattice vectors and u_j connecting the two different sublattices. We emphasize again that a finite displacement field introduces a finite gap Δ and also modifies the hopping $t_n^{AA/BB}$ for the two different sublattices. A general expression for the intrasublattice dispersion and the interlayer tunneling read:

$$\epsilon_{ka} = 2\sum_n t_n^a \sum_{j\in R_n} \cos k\cdot(x_{nj}a_1 + y_{nj}a_2), \quad t_k = \sum_n t_n^{AB} \sum_{j\in R_n} e^{ik\cdot(u_1 + x_{nj}a_1 + y_{nj}a_2)}, \tag{S23}$$

with R_n identifying the n-th shell of lattice sites.

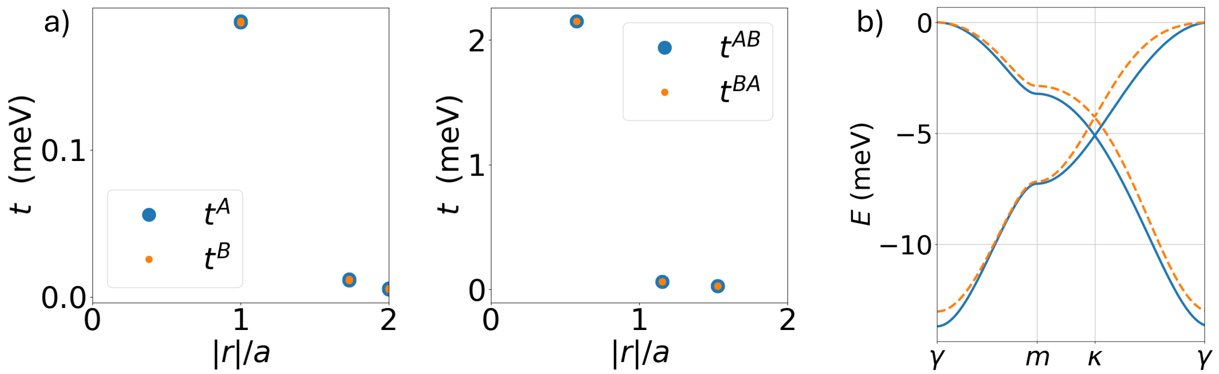

FIG. S8. Panel a) shows the intralayer $t^{A/B}$ (left) and interlayer $t^{AB/BA}$ (right) hopping amplitudes as a function of the distance between sites $|r|$. Panel b) shows a comparison between the continuum model and tight-binding bands obtained including only hopping between sites up to $|r| \leq a$. Results are obtained setting $\theta = 2°$.

The leading hopping amplitude is the intersublattice n.n. amplitude t_1^{AB}, which including the sublattice potential Δ and the n.n. repulsion $V = e^2/(4\pi\epsilon_0\epsilon a)$ define the minimal model investigated via exact diagonalization. The ratio between V and t is shown in the right panel of Fig. S6.

5. Strong coupling Hamiltonian: spin model at filling 1

Additional insights into the magnetic properties can be gained through the tight-binding model (S22) derived from the continuum model. In the small twist-angle regime, the interacting tight-binding model reads:

$$H = t \sum_{\langle r,r' \rangle} c_r^\dagger c_{r'} + t' \sum_{\alpha=A,B} \sum_{\langle r,r' \rangle \in \alpha} c_r^\dagger c_{r'} + U \sum_r n_{r\uparrow} n_{r\downarrow} + V \sum_{\langle r,r' \rangle} n_r n_{r'}, \tag{S24}$$

where t is the nearest-neighbor and t' the next-nearest neighbor hoppings with $t'/t = 0.09$ and $t = 2$meV for $\theta = 2°$. The hopping parameters and a comparison between the tight-binding Hamiltonian including t and t' (S24), and continuum model band structures are shown in Fig. S8.

In the sublattice-polarized insulator at $\nu = 1$, where the charge degrees of freedom are localized on a triangular lattice—either sublattice A or B—the effective spin-exchange Hamiltonian takes the form:

$$H_{\rm spin} = J \sum_{\langle r,r' \rangle \in \alpha} \mathbf{S_r} \cdot \mathbf{S_{r'}}. \tag{S25}$$

Here, $J = 4t'^2/U - t't^2/(V + \Delta/2)^2$, where the first (antiferromagnetic) contribution arises from the leading exchange processes, including superexchange, while the second (ferromagnetic $t' > 0$ in Fig. S8) term originates from loop exchange processes [4, 7]. In the small twist angle regime and for screened Coulomb interactions, where U constitutes the dominant energy scale and $t'/t \leq 0.1$, leading to $J < 0$.

Appendix B: Exact Diagonalization Calculations of the lattice model

We perform exact diagonalization calculations of the hexagonal lattice model with parameters t, V and Δ:

$$H = -t \sum_k \left(f_k \psi_{kA}^\dagger \psi_{kB} + h.c. \right) - \Delta \frac{\psi_{kA}^\dagger \psi_{kA} - \psi_{kB}^\dagger \psi_{kB}}{2} + \frac{V}{N} \sum_{\{k\}} \delta_{k_1+k_2,k_3+k_4} f(k_3 - k_2) \psi_{k_1A}^\dagger \psi_{k_2B}^\dagger \psi_{k_3B} \psi_{k_4A}, \tag{S26}$$

the form factor is $f_k = \sum_{j=1,2,3} e^{ik\cdot u_j}$ and $u_j = e^{2i\pi(j-1)/3}/\sqrt{3}$ in complex notation. These parameters can be derived explicitly from the knowledge of the Wannier orbitals. Additionally, we have introduced the fermionic operators:

$$\psi_{kA} = \frac{1}{\sqrt{N}} \sum_{r\in A} e^{ik\cdot r} f_r, \quad \psi_{kB} = \frac{1}{\sqrt{N}} \sum_{r\in A} e^{ik\cdot(r+u_1)} f_{r+u_1}. \tag{S27}$$

We performed numerical diagonalization for system sizes 3×4 (24 sites in total) with periodic boundary conditions. In our numerical simulations for 12 sites we perform full diagonalization of the model.

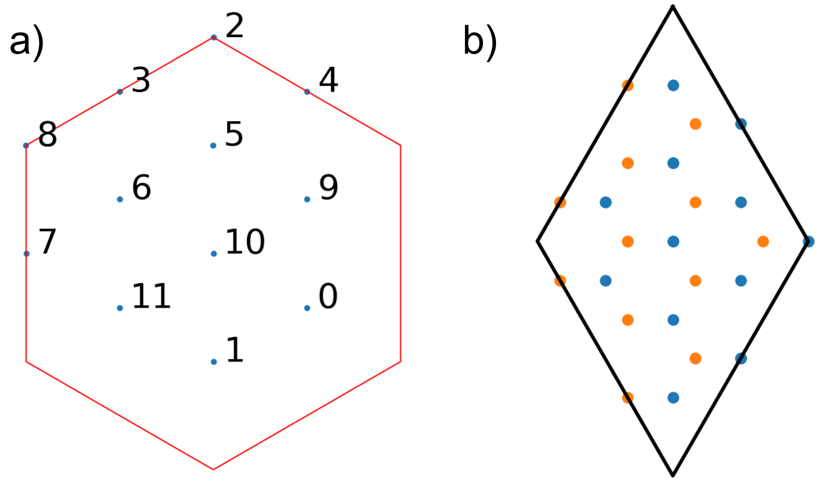

FIG. S9. Momentum grids in the BZ for 12 corresponding to 24 sites in total.

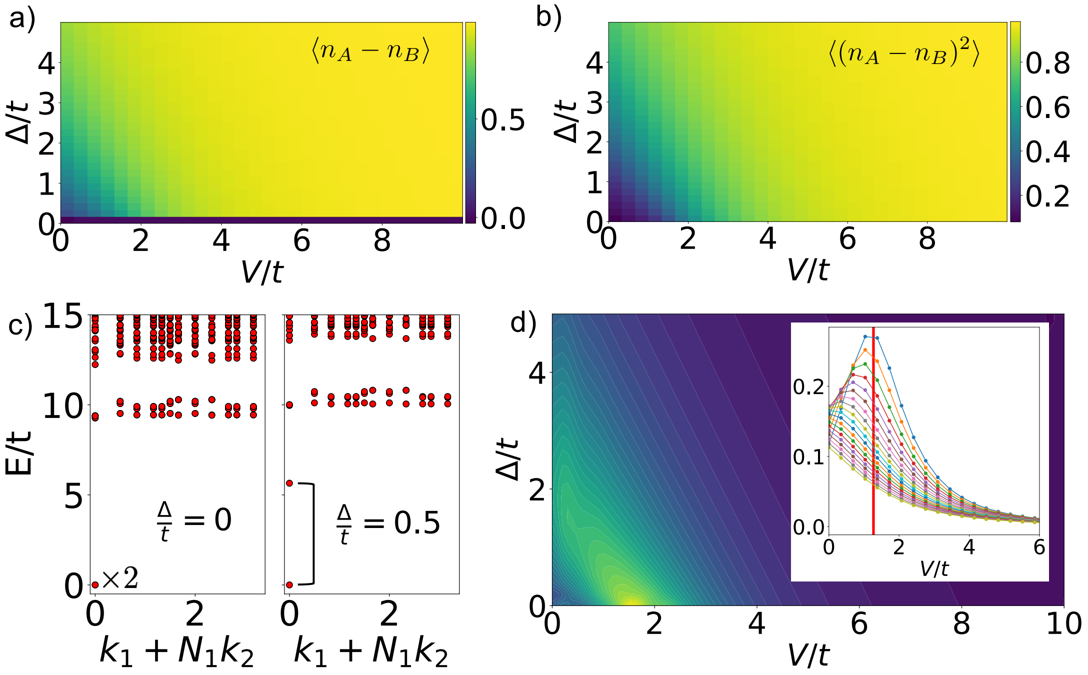

FIG. S10. Panel a) and b) show $\langle n_A - n_B \rangle$ and $\langle (n_A - n_B)^2 \rangle$ in a relevant range of Δ/t and V/t. Panel c) illustrates the many-body spectrum for $V/t = 5$ and two different values of $\Delta/t = 0$ (left) and $\Delta/t = 0.5$ (right). Panel d) shows the derivative of $\langle (n_A - n_B)^2 \rangle$ with respect to Δ at fixed Δ.

1. Incompressible state at filling $\nu = 1$

In this section, we detail the properties of the parent state of the superconductor. This state is a sublattice-polarized insulator, where the symmetry between the two sublattices is broken. The symmetry breaking occurs spontaneously at $\Delta = 0$ and explicitly for any finite Δ. Fig. S10a) and S10b) show the expectation values $\langle n_A - n_B \rangle$ and $\langle (n_A - n_B)^2 \rangle$ with $n_{A/B} = N_{A/B}/N$. The increase of $\langle (n_A - n_B)^2 \rangle$ signals the system's approach toward a strongly correlated sublattice insulator. Fig. S10c) displays the many-body spectrum at filling $\nu = 1$ for two different values of Δ/t. The two-fold degeneracy of the ground state at $\Delta = 0$ signals the degeneracy of the two sublattice insulating states with opposite sublattice polarization that are connected by C_{2y}. Introducing Δ lifts the degeneracy opening a gap of approximately $N_s\Delta$ growing extensively with system size. Finally, Fig. S10d) shows the derivative of $(n_A - n_B)^2$ with respect to Δ. The inset of Fig. S10d) shows a peak at $V_c/t = 1.3$ (solid red line) that we interpret as the location of

the Ising Gross-Neveu critical point [8] at zero displacement field.

2. Computing properties of the $2e$ bound state: effective mass, mean square radius and binding energy

In this section we detail the evaluation of the binding energy, effective mass and the mean square radius. To simplify the notation we define E_p the ground state energy in the sector with $N+p$ particles. The binding energy per particle is defined as:

$$E_b = E_{1e} - E_{2e}/2, \tag{S28}$$

where:

$$E_{2e} = E_{+2} - E_0, \quad E_{1e} = E_{+1} - E_0, \tag{S29}$$

E_0 and E_{+2} lies in the sector with center of mass momentum $Q = 0$, while E_{+1} is defined as:

$$E_{+1} \equiv \min_{Q} E_{+1}(Q), \tag{S30}$$

where the minimum is found scanning over different momentum sectors. In agreement with perturbative results, we find that the minimum in the particle sector 1 is always located at K/K'. Finally, the charge gap is defined as:

$$E_{\text{gap}} = E_{+1} + E_{-1} - 2E_0, \tag{S31}$$

We compute the mass of the pair looking at the dispersion in the many-body space with 2 particles:

$$\frac{1}{m_B} = \left.\frac{\nabla_Q^2 E_{+2}(Q)}{2}\right|_{Q=0}, \tag{S32}$$

where the isotropy of the mass follows from the C_{3z} symmetry of the theory. Given the D_6 symmetric cluster in Fig. S9 the second derivative (S32) can be approximated employing the first shell of momenta:

$$\frac{1}{m_b} = \frac{1}{2}\sum_l \frac{E_{+2}(Q = \Delta k_l) - E_{+2}(\Gamma)}{\sum_{j=1}^{3}(1 - \cos \Delta k_l \cdot a_j)}. \tag{S33}$$

Finally, the mean square radius $\langle r^2 \rangle$ is obtained by first computing the two particle bound state wavefunction

$$\Psi_{2e}(x + \Delta x, x) = \langle \Psi_N | f_{x+\Delta x} f_x | \Psi_{N+2} \rangle, \tag{S34}$$

over the ground state obtained via exact diagonalization. The latter average value reads:

$$\langle \Psi_N | f_{x+\Delta x} f_x | \Psi_{N+2} \rangle = \frac{1}{N}\sum_{kk'} e^{ik\cdot(x+\Delta x) + ik'\cdot x} \langle \Psi_N | \psi_{k\alpha} \psi_{k'B} | \Psi_{N+2} \rangle, \tag{S35}$$

where x belongs to the B sublattice, $\alpha = A, B$ depending on the sublattice site and momentum conservation selects the amplitudes with $k + k' = 0$ mod reciprocal lattice vectors $\mod [l_j \cdot (k + k'), 2\pi] = 0$. Fig. S11a) shows the $2e$ wavefunction where the size of the dots represents the absolute value of the wavefunction and the colorcode the phase. Finally, we observe that the wavefunction is finite but small in the A-sublattice, with average occupation number $\langle n_A \rangle \approx 1$. Furthermore, the wave function transforms as an A_2 irreducible representation of the D_3 point group, invariant under C_{3z} and odd under C_{2y}, lattice version of an f-wave [9–11]. We quantify the spread through the mean square radius $\langle r^2 \rangle$ defined as

$$\langle r^2 \rangle = \frac{\sum_{\Delta r} |\Psi_{2e}(r + \Delta r, r)|^2 \overline{\Delta r}^2}{\sum_{\Delta r} |\Psi_{2e}(r + \Delta r, r)|^2} \tag{S36}$$

where $\overline{\Delta r}$ is the distance from r module (L_1, L_2), i.e. it is invariant under a shift of $\Delta r \to \Delta r + L_{1/2}$ with $L_{1/2}$ dimension of the cluster setting the largest length scale resolved in the numerics. Fig. S11b) illustrates the mean-square radius computed numerically.

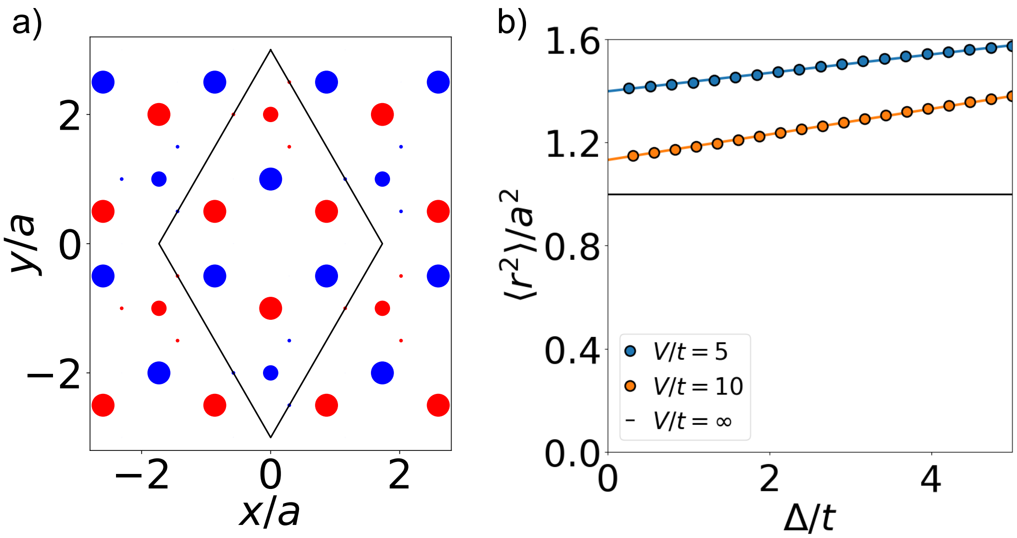

FIG. S11. Panel a) $2e$ bound state wavefunction on a torus (L_1, L_2) including 12 unit cells for $V/t = 10$ and $\Delta/t = 0.8$. The size of the dots show $|\Psi_{2e}|$ and the color represents the phase blue (0) and red (π). Panel b) presents the mean square radius, with dotted lines showing the power-law fit $\langle r^2\rangle/a^2 = A\Delta/t + B$ obtained in the long wavelength limit.

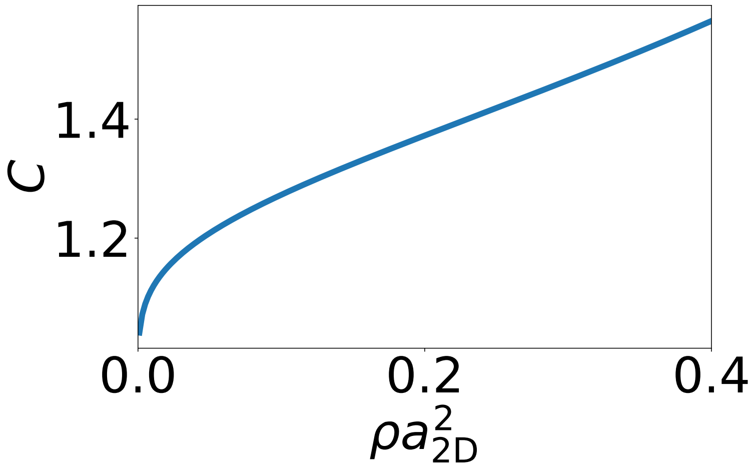

FIG. S12. Evolution of the coefficient C as a function of ρa_{2D}^2.

From the evaluation of the effective mass m_b and the mean square radius $\langle r^2\rangle$, we estimate the optimal critical temperature as [12–17]:

$$T_c = C\frac{\hbar^2\rho}{m_b}, \tag{S37}$$

where $\rho = 1/(\pi\langle r^2\rangle)$. In Eq. S37, the coefficient C depends on the repulsive interaction between the bosons. Specifically, we have [12–14, 18]:

$$C = \frac{2\pi}{\log(380/4\pi) + \log\log(1/\rho a_{\mathrm{2D}}^2)}, \tag{S38}$$

where ρ is the density of bosons and a_{2D} is the 2D scattering length. The evolution of C is shown in Fig. S12, and due to the double logarithmic behavior, it exhibits only a weak dependence on ρa_{2D}^2. We set $C \approx 2\pi/\log(380/4\pi)$ in our calculations. Fig. S13 shows the evolution of T_c as a function of Δ for different values of V/t. The shaded gray area highlights the region where an attraction between two excitonic Cooper pairs emerges. In this regime, at finite density, the system's properties are governed by a complex interplay between charge density waves and superconductivity, which will be explored in future studies.

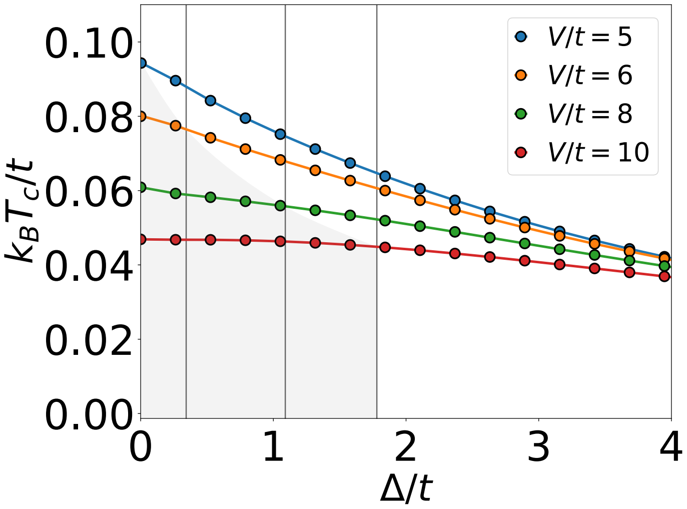

FIG. S13. Optimal critical temperature computed from ED simulations.

Appendix C: Exact results in the $V \to \infty$ limit

In this section we present exact results on the spectrum of charge excitations of the model. The $V \to \infty$ constraint selects only intersublattice excitations preserving the number of nearest-neighbor intersublattice pairs. As a result, in this limit, charged excitations are localized and the exact spectrum of eigenstates is found by diagonalizing the Hamiltonian on a finite size cluster.

1. charge-$2e$ excitation

For strong repulsion, a pair of doped carriers is confined to a triangular cluster and exhibits a finite binding energy, making the charge-$2e$ complex energetically favorable compared to two separate charge-e excitations. The binding energy originates from the charge-transfer exciton connecting the $2e$ state with the trimer configuration. The Hamiltonian describing the four-sites cluster reads:

$$H_{2e} = \Delta \sum_{j=1}^{3} (f_j^\dagger f_j - 2) - t \sum_{j=1}^{3} \left(f_j^\dagger f_0 + h.c. \right), \tag{S39}$$

with four sites and total charge $Q = 3$. We readily find two zero energy configurations:

$$|E_1\rangle = \frac{f_1^\dagger f_2^\dagger - f_2^\dagger f_3^\dagger}{\sqrt{2}} f_0^\dagger |0\rangle, \quad |E_2\rangle = \frac{f_1^\dagger f_2^\dagger + f_1^\dagger f_3^\dagger}{\sqrt{2}} f_0^\dagger |0\rangle, \tag{S40}$$

forming a two-dimensional irreducible representation, $C_{3z} |E_1\rangle = |E_2\rangle - |E_1\rangle$ and $C_{3z} |E_2\rangle = -|E_1\rangle$. In addition, we find two C_{3z} invariant configurations $\prod_{j=1}^{3} f_j^\dagger |0\rangle$ and $\left(\sum_{j=1}^{3} f_j^\dagger f_{j+1}^\dagger \right) f_0^\dagger |0\rangle / \sqrt{3}$. Projecting H_{2e} in this two-dimensional subspace we find that the ground state of the model is the bonding configuration:

$$\begin{aligned} |A\rangle &= \sqrt{\frac{1}{2} - \frac{\Delta}{4\sqrt{\Delta^2/4 + 3t^2}}} \prod_{j=1}^{3} f_j^\dagger |0\rangle + \sqrt{\frac{1}{2} + \frac{\Delta}{4\sqrt{\Delta^2/4 + 3t^2}}} \left(\frac{1}{\sqrt{3}} \sum_{j=1}^{3} f_j^\dagger f_{j+1}^\dagger \right) f_0^\dagger |0\rangle, \\ E_A &= \frac{\Delta}{2} - \sqrt{\frac{\Delta^2}{4} + 3t^2}, \end{aligned} \tag{S41}$$

which belongs to the A_2 irreducible representation of the point group of the crystal. We conclude that the binding energy reads:

$$E_b = E_{1e} - E_{2e}/2 = \frac{1}{2}\sqrt{\frac{\Delta^2}{4} + 3t^2} - \frac{\Delta}{4} \geq 0. \tag{S42}$$

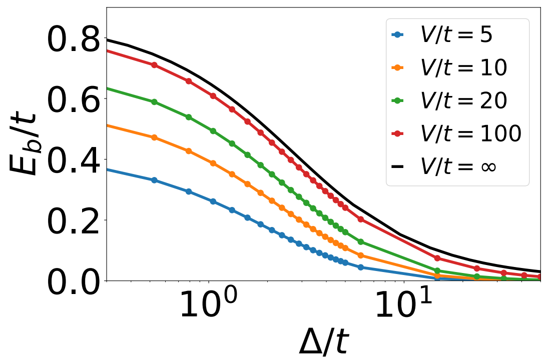

FIG. S14. Binding energy as a function of Δ/t for different V/t. The solid black line shows the binding energy for $V \to \infty$.

We emphasize that trimer charge fluctuations serve as the glue that enables the formation of the bound state. Before proceeding, we present the evolution of the binding energy as a function of Δ/t for various values of V/t in Fig. S14. Increasing V/t the results obtained with ED approaches the asymptotic value in Eq. (S42).

Before concluding this section, we show that the pair wavefunction belongs to the A_2 irrep of C_{3v} computing explicitly the symmetry eigenvalues. To this end, we consider the charge-$2e$ wavefunction $\Psi_{2e}(r+\Delta r, r)$ (S34) in the $V \to \infty$ limit, where we obtain the exact expression:

$$\Psi_{2e}(r+\Delta r, r) = \langle \Phi_0 | f_{r+\Delta r} f_r | \Phi_2 \rangle = \alpha \sum_{i=1}^{3} \frac{\delta_{\Delta r, u_{i+1}-u_i} - \delta_{\Delta r, u_i - u_{i+1}}}{\sqrt{3}}, \tag{S43}$$

with $r \in B$, $i = 1, 2, 3$, and the $2e$ bound state is confined within a single unit cell $\langle r^2 \rangle = a^2$. Under a C_{3z} rotation around r, the pair wavefunction is invariant:

$$\Psi_{2e}(r + C_{3z}\Delta r, r) = \alpha \sum_{i=1}^{3} \frac{\delta_{\Delta r, u_i - u_{i-1}} - \delta_{\Delta r, u_{i-1} - u_i}}{\sqrt{3}} = \Psi_{2e}(r+\Delta r, r). \tag{S44}$$

On the other hand, under M_y, we find

$$\begin{aligned}\Psi_{2e}(r + M_y\Delta r, r) &= \alpha \sum_{i=1}^{3} \frac{\delta_{M_y\Delta r, u_{i+1}-u_i} - \delta_{M_y\Delta r, u_i - u_{i+1}}}{\sqrt{3}} = \alpha \sum_{i=1}^{3} \frac{\delta_{\Delta r, u_i - u_{i+1}} - \delta_{\Delta r, u_{i+1} - u_i}}{\sqrt{3}} \\ &= -\Psi_{2e}(r+\Delta r, r),\end{aligned} \tag{S45}$$

demonstrating that Ψ_{2e} is odd under the mirror symmetry M_y. Consequently, Ψ_{2e} belongs to the A_2 irrep of C_{3v}, lattice version of a f-wave. The property persists for finite value of V/t, as displayed in Fig. S11 by the numerical evaluation of Ψ_{2e}. Finally, associated to the charge-$2e$ bound state (S41) we introduce a new emergent quasiparticle $b_r^\dagger$ located at r with bosonic statistics:

$$b_r^\dagger = \sqrt{1 - |\alpha|^2} \prod_{j=1}^{3} f_{r'_j}^\dagger f_r + \frac{\alpha}{\sqrt{3}} \sum_{j=1}^{3} f_{r'_j}^\dagger f_{r'_{j+1}}^\dagger, \tag{S46}$$

where r'_j ($j = 1, 2, 3$) are the lattice sites $r + u_j$, which are nearest neighbors of site r. Acting on the sublattice-polarized insulator $|\Phi_0\rangle$ generates the configuration $|\Phi_2(r)\rangle = b_r^\dagger |\Phi_0\rangle$.

To quantitatively assess the agreement between the exact charge-$2e$ wavefunction and the ground state obtained via exact diagonalization of the full many-body Hamiltonian, we evaluate the fidelity, defined as:

$$\mathcal{F} = |\langle \Psi_{2e} | \Phi_2(Q=0) \rangle|, \tag{S47}$$

where $|\Phi_{2e}(Q)\rangle$ is the exact infinite V two-particle wavefunction with center of mass momentum Q:

$$|\Phi_2(Q)\rangle = \sum_r e^{-iQ\cdot r} b_r^\dagger |\Phi_0\rangle / \sqrt{N}, \tag{S48}$$

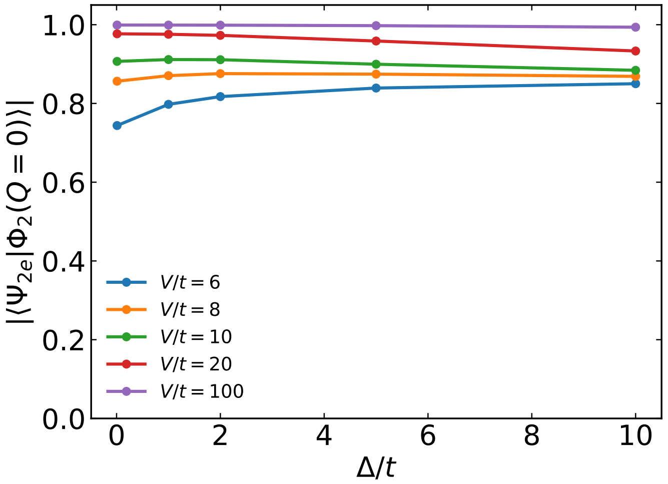

FIG. S15. Evolution of the fidelity as a function of Δ/t for different values of V/t.

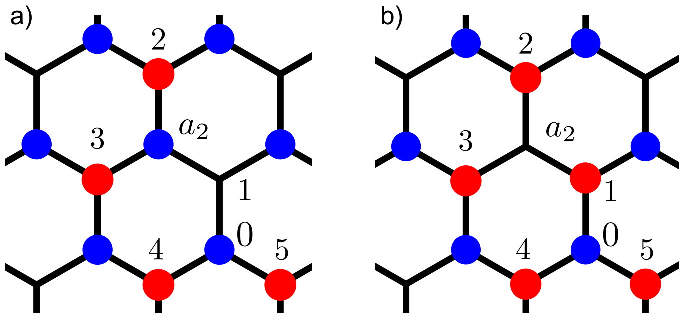

FIG. S16. Panel a) shows one of the 5 configurations corresponding to adding 4 carriers in 5 sites $j = 1, \cdots, 5$. Panel b) shows the configuration obtained exciting a trimer centered around a_2.

with $|\Phi_0\rangle = \prod_k \psi^\dagger_{kA} |0\rangle$. The $Q = 0$ ground state takes the form:

$$|\Phi_2(0)\rangle = \Bigg[\frac{\alpha}{\sqrt{3N}} \sum_{kk'} \sum_G \delta_{k+k',G} \left(\sum_{j=1}^{3} e^{-i\left(k\cdot u_j + k'\cdot u_{j+1}\right)} \right) \psi^\dagger_{kB}\psi^\dagger_{k'B} + \sqrt{\frac{1-\alpha^2}{N^3}} \sum_{k_1\cdots k_4} e^{-i(k_1\cdot r_1 + k_2\cdot r_2 + k_3\cdot r_3)} \psi^\dagger_{k_1B}\psi^\dagger_{k_2B}\psi^\dagger_{k_3B}\psi_{k_4A} \sum_G \delta_{G,k_4-k_1-k_2-k_3} \Bigg] |\Phi_0\rangle \, . \tag{S49}$$

Fig. S15 shows the fidelity $\mathcal{F}$ obtained computing the overlap between the exact ground state for one doped excitonic Cooper pair $|\Phi_2(0)\rangle$ and the numerical ground state $|\Psi_{2e}\rangle$ at finite V/t.

2. charge-$4e$ excitation

We now calculate the energy of a charge-$4e$ composed by two charge-$2e$ pairs considering the case where the Cooper pairs are centered around neighboring sites and next to nearest-neighbor sites.

a. Nearest-Neighbor Repulsion

We start considering two Cooper pairs centered around $r = 0$ and $r = a_2$ in Fig. S16 and showing that as a result of the Pauli exclusion principle we find a repulsive interaction between the two. We have 5 configurations with 4 carriers

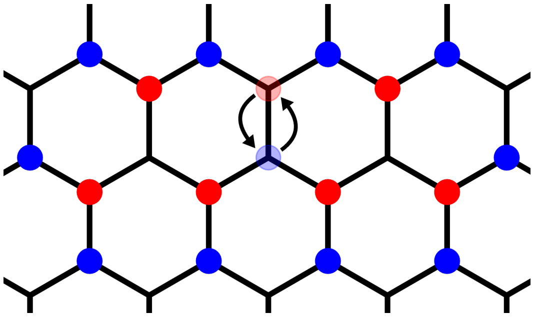

FIG. S17. Two Cooper pairs centered around two next-n.n. sites, i.e. positioned at r and $r + 2a_1$, lower their energy by a virtual exciton involving the intermediate center at $r + a_1$.

in the B-sublattice:

$$|\{j\}\rangle = f^\dagger_{j_1} f^\dagger_{j_2} f^\dagger_{j_3} f^\dagger_{j_4} f^\dagger_0 f^\dagger_{a_1} |0\rangle \,, \tag{S50}$$

with $\{j\}$ denotes the five independent ways to select four labels from a set of five sites. Additionally, we have two excited states corresponding to a single trimer excitation centered around a_2 and 0, respectively, with energy Δ:

$$|a_2\rangle = \prod_{j=1}^{5} f^\dagger_j f^\dagger_0 |0\rangle \,, \quad |0\rangle = \prod_{j=1}^{5} f^\dagger_j f^\dagger_{a_2} |0\rangle \,. \tag{S51}$$

Computing the overlaps between the different configurations we find the Hamiltonian on nearest-neighbors:

$$H_{4e} = \begin{pmatrix} 0 & 0 & 0 & 0 & 0 & -t & t \\ 0 & 0 & 0 & 0 & 0 & 0 & -t \\ 0 & 0 & 0 & 0 & 0 & 0 & t \\ 0 & 0 & 0 & 0 & 0 & t & 0 \\ 0 & 0 & 0 & 0 & 0 & -t & 0 \\ -t & 0 & 0 & t & -t & \Delta & 0 \\ t & -t & t & 0 & 0 & 0 & \Delta \end{pmatrix} . \tag{S52}$$

The ground state energy is $E_{gs} = \Delta/2 - \sqrt{8t^2 + \Delta^2}/2 > 2E_A$ (S41) implying a net repulsive interaction between Cooper pairs on n.n. sites.

b. Next to Nearest-Neighbor Attraction

We consider two Cooper pairs, one positioned at $r = 0$ and the other at $r = 2a_1$. Employing the bosonic operator $b^\dagger_r$ (S46) the corresponding configuration is:

$$|\Phi_4(r, r + 2a_1)\rangle = b^\dagger_r b^\dagger_{r+2a_1} |\Phi_0\rangle \,. \tag{S53}$$

The Coulomb restricted tunneling $T_0 = -t\mathbb{P}_{12} \sum_{\langle r,r'\rangle} f^\dagger_r f_{r'} \mathbb{P}_{12}$ with 12 counting the number of n.n. bonds introduces quantum dynamics in the low-energy manifold which lowers the energy of the $4e$ charge complex with respect to the one of two isolated Cooper pairs. One among many processes is drawn in Fig. S17 where the middle site between the two Cooper pairs is resonating along u_1. We anticipate that in the infinite V regime the Cooper pairs form a crystal (bosonic CDW) on n.n.n. sites with enlarged unit cell $(3a_1, 2a_2)$.

Fig. S18 shows exact diagonalization (ED) results for $E_{2e} - E_{4e}/2$, where $E_{4e} = E_{+4} - E_0$. At finite V/t, we find that the interaction between excitonic Cooper pairs is repulsive for $V/t < 5$. For larger interaction strengths, the boson b_r develops a net attractive interaction, leading to the formation of a four-body bound state. In this regime, increasing Δ/t tunes the interaction between Cooper pairs from attractive to repulsive.

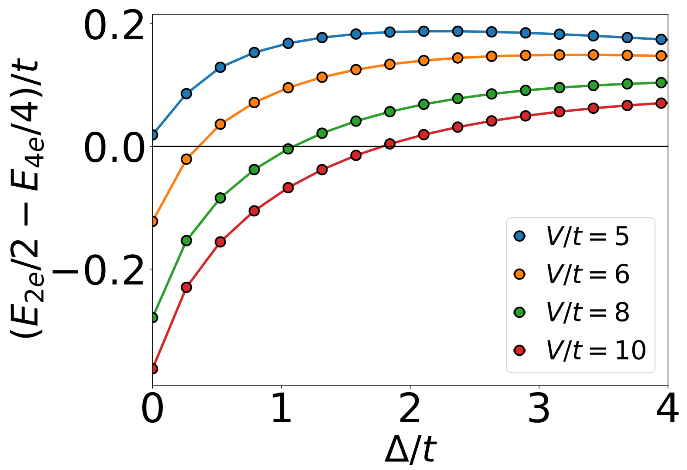

FIG. S18. Energy difference $E_{2e}/2 - E_{4e}/4$ indicating a tendency to form a four-body charge complex when $E_{2e}/2 - E_{4e}/4 < 0$ for different values of V/t.

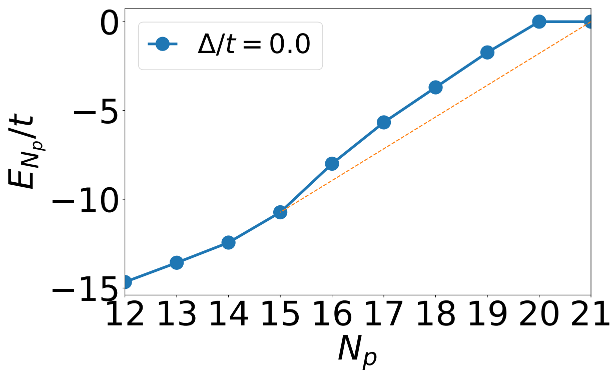

FIG. S19. Ground state energy as a function of the number of particles N_p obtained performing ED calculations on a cluster of 42 sites.

3. Infinite coupling model

In this section, we discuss the ground state properties obtained for larger doping in the limit $V \to \infty$. Exploiting the particle-hole symmetry of the model, we focus on the regime $N_p < N$ (hole-doped) with N number of unit cells. In this regime, the Hamiltonian (S26) takes the simple form:

$$H_\infty = -t \sum_{\langle r,r' \rangle} P_r f_r^\dagger f_{r'} P_{r'} + \Delta N_B, \tag{S54}$$

where hopping is permitted only between configurations that avoid nearest-neighbor occupancies:

$$P_r = \prod_{r' \text{ next to } r} (1 - n_{r'}). \tag{S55}$$

The model features a single dimensionless parameter Δ/t and the constraint in the hopping makes the problem strongly interacting. Fig. S19 shows the ground state energy for different numbers of carriers in the hole-doped regime. The concave behavior observed in the energy curve between 15 and 21 provides evidence for a tendency toward phase separation within this doping range.

Appendix D: Strong coupling perturbation theory

Here, we present the first-order correction in t/V for the large V regime. We discuss the corrections to the ground state energy for filling factors N, $N+1$ and $N+2$, binding energy and effective mass. Perturbation theory in small t/V is performed organizing the spectrum of the model in sectors with a fixed number n.n. occupied sites M. The Hamiltonian decomposes as $H = H_0 + H'$ with H_0 block diagonal in the subspace M, i.e. it does not mix different subspaces:

$$H_0 = \sum_M H_M, \quad H_M = \Delta N_B + MV. \tag{S56}$$

The off-diagonal contribution instead mixes the subspace M with $M+q$:

$$H' = \sum_M \sum_q T_{q,M}, \quad T_{q,M} = -t\mathbb{P}_{M+q} \sum_{\langle r,r'\rangle} f_r^\dagger f_{r'} \mathbb{P}_M, \tag{S57}$$

where $q = \pm 1, \pm 2$ corresponds to the addition/removal of q n.n. occupied sites. To connect with previous works [9, 19], we observe that the action of T_1 creates a polaron and T_2 creates a dipole. Finally, we emphasize that $T_{0,M}$ preserves $\sum_{\langle r,r'\rangle} n_r n_{r'}$ which is equal to MV but does not commute with $N_A - N_B$, thereby introducing quantum dynamics within each sector M.

1. Schrieffer-Wolff transformation: effective Hamiltonian upon doping the sublattice polarized state

In this section, we clarify the connection between the many-body perturbation theory, and the Schrieffer-Wolff transformation in the many-body space. Without losing generality, we consider charge sector of $N + p$ particles with N number of sites and p extra doped carriers. Moreover, for simplicity we consider the case $\Delta = 0$, where for large V the ground state spontaneously polarize in one of the two sublattices. Within each sector, we introduce the basis of states $|\Phi_{p,nM}\rangle$ where p denotes the sector with p extra doped carriers ($p = 0, 1, 2$), M the number of n.n. occupied sites and n the principal quantum number. By definition the unperturbed Hamiltonian projected in the particle sector p reads:

$$H_0 = \sum_M \sum_n E_{nM} |\Phi_{p,nM}\rangle \langle \Phi_{p,nM}|, \tag{S58}$$

while H' is off-diagonal and has the form:

$$H' = \sum_M \sum_q \sum_{nm} (T_{q,M})_{nm} |\Phi_{p,nM+q}\rangle \langle \Phi_{p,mM}|. \tag{S59}$$

By definition we have:

$$[H_0, T_{q,M}] = qVT_{q,M}. \tag{S60}$$

Moreover, we have the relations:

$$\begin{aligned} &[H_0, T_{q_1,M_1} T_{q_2,M_2}] = (q_1 + q_2)VT_{q_1,M_1} T_{q_2,M_2}, \\ &[H_0, [T_{q_1,M_1}, T_{q_2,M_2}]] = (q_1 + q_2)V[T_{q_1,M_1}, T_{q_2,M_2}], \end{aligned} \tag{S61}$$

which directly follows from the properties of the commutator. Notice that for any particle sector p the lowest energy configuration has a number of n.n. occupied sites $M = zp$ with $z = 3$ coordination of the lattice. Moreover, for $p = 0$ the ground state is only two-fold degenerate corresponding to a fully polarized sublattice insulator. On the other hand, for $p = 1, 2$ the ground state is extensively degenerate.

The Schrieffer-Wolff transformation consists of introducing the antiunitary operator S, $S^\dagger = -S$, leading to the transformed Hamiltonian:

$$\bar{H} = e^S H e^{-S}. \tag{S62}$$

Employing Baker-Campbell-Hausdorff identity to third order in S, we have:

$$\bar{H} = H + [S, H] + \frac{1}{2}[S, [S, H]] + \frac{1}{3!}[S, [S, [S, H]]] + \cdots. \tag{S63}$$

Our task is to define S which order by order in (t/V) allows to remove terms off-diagonal in the number of n.n. occupied sites M. Specifically, the transformation $S^{(k)} \sim \mathcal{O}[(t/V)^k]$ removes all off-diagonal terms of order $(t/V)^{k-1}$. To start with we define, the Hamiltonian at stage $k=1$ as:

$$\bar{H}^{(1)} \equiv H = H_0 + H', \tag{S64}$$

and $S^{(0)} = 1$ is the identity. At stage $k=2$, we have:

$$\bar{H}^{(2)} = e^{S^{(1)}} H e^{-S^{(1)}} = H + [S^{(1)}, H_0] + [S^{(1)}, H'] + \frac{1}{2}[S^{(1)}, [S^{(1)}, H_0]] + \mathcal{O}\left(\frac{t^3}{V^2}\right). \tag{S65}$$

Imposing the conditions $[S^{(1)}, H_0] = -\sum_{q\neq 0}\sum_M T_{q,M}$, we find:

$$S^{(1)} = \sum_M \sum_{q\neq 0} \sum_{nm} \frac{(T_{q,M})_{nm}}{qV} \left|\Phi_{p,nM+q}\right\rangle \left\langle \Phi_{p,nM}\right|, \tag{S66}$$

where we have introduced the notation $(T_{q,M})_{nm} \equiv \langle \Phi_{p,nM+q}|T_{q,M}|\Phi_{p,mM}\rangle$. As a result, the second order Hamiltonian $H^{(2)}$ reads:

$$\bar{H}^{(2)} = H_0 + \sum_M T_{0,M} + \sum_{M_1 M_2}\sum_{q\neq 0} \frac{[T_{q,M_1}, T_{0,M_2}]}{qV} + \frac{1}{2}\sum_{M_1 M_2}\sum_{q_1 q_2 \neq 0} \frac{[T_{q_1,M_1}, T_{q_2,M_2}]}{q_1 V} + \mathcal{O}\left(\frac{t^3}{V^2}\right). \tag{S67}$$

Projecting the Hamiltonian in the lowest energy manifold with number of n.n. occupied sites $M = zp$ we obtain:

$$\mathcal{H}^{(p)} = H_{zp} - \sum_{q=1}^{2} \frac{T^\dagger_{q,zp} T_{q,zp}}{qV} + \mathcal{O}\left(\frac{t^3}{V^2}\right). \tag{S68}$$

where $H_{zp} = H_0 + T_{0,zp}$.

a. *Next-to-leading order corrections*

To obtain an accurate expression for the effective mass of the charge-$2e$ excitation and the binding energy valid to larger values of t/V, we extend our perturbation theory up to order t^3/V^2. To this aim we introduce $S^{[2]} \sim (t/V)^2$ which removes off-diagonal terms of order t/V:

$$\begin{aligned}\bar{H}^{(3)} =& e^{S^{(2)}} H e^{-S^{(2)}} = H_0 + \sum_M T_{0,M} + \sum_{M_1 M_2}\sum_{q\neq 0} \frac{[T_{q_1,M_1}, T_{0,M_2}]}{q_1 V} + \frac{1}{2}\sum_{M_1 M_2}\sum_{q_1 q_2 \neq 0} \frac{[T_{q_1,M_1}, T_{q_2,M_2}]}{q_1 V} \\ &+ [S^{[2]}, H_0] + [S^{[2]}, H'] + \sum_M \frac{[S^{(1)}, [S^{(1)}, T_{0,M}]]}{2} + \sum_{q\neq 0}\sum_M \frac{[S^{(1)}, [S^{(1)}, T_{q,M}]]}{3},\end{aligned} \tag{S69}$$

where $S^{(2)} = S^{[1]} + S^{[2]}$ with $S^{[1]} = S^{(1)}$ and $S^{[2]}$ such that:

$$[S^{[2]}, H_0] = -\sum_{M_1 M_2}\sum_{q_1 \neq 0} \frac{[T_{q_1,M_1}, T_{0,M_2}]}{q_1 V} - \frac{1}{2}\sum_{M_1 M_2}^{M_1+q_1\neq M_2}\sum_{q_1 q_2 \neq 0} \frac{[T_{q_1,M_1}, T_{q_2,M_2}]}{q_1 V}. \tag{S70}$$

$S^{[2]}$ is obtained employing the identity (S60) and is composed by the sum of two contributions:

$$S^{[2]} = \sum_{M_1 M_2}\sum_{q_1\neq 0} \frac{[T_{q_1,M_1}, T_{0,M_2}]}{(q_1 V)^2} + \frac{1}{2}\sum_{M_1 M_2}^{M_1+q_1\neq M_2}\sum_{q_1 q_2\neq 0} \frac{[T_{q_1,M_1}, T_{q_2,M_2}]}{q_1(q_1+q_2)V^2}. \tag{S71}$$

The resulting third order Hamiltonian reads:

$$\begin{aligned}\bar{H}^{(3)} =& H_0 + \sum_M T_{0,M} + \frac{1}{2}\sum_{M_1 M_2}^{M_1+q_1=M_2}\sum_{q_1 q_2\neq 0} \frac{[T_{q_1,M_1}, T_{q_2,M_2}]}{q_1 V} \\ &+ [S^{(2)}, H'] + \sum_M \frac{[S^{(1)}, [S^{(1)}, T_{0,M}]]}{2} + \sum_{q\neq 0}\sum_M \frac{[S^{(1)}, [S^{(1)}, T_{q,M}]]}{3} + \mathcal{O}\left(\frac{t^4}{V^3}\right),\end{aligned} \tag{S72}$$

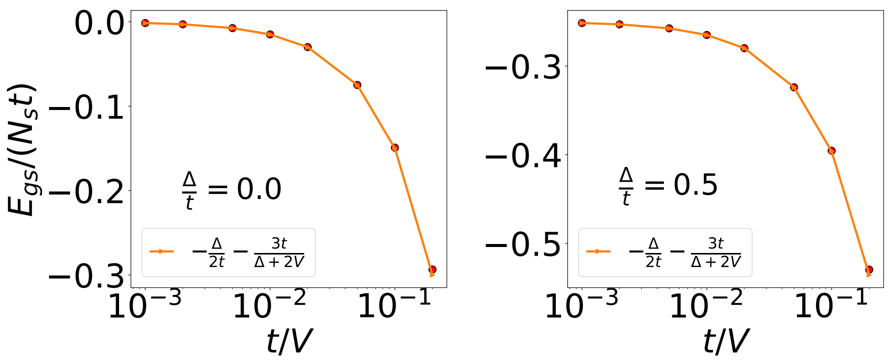

FIG. S20. Left and right panel shows the evolution of the many-body ground state energy per particle as a function of t/V obtained setting $\Delta = 0$ in the left panel and $\Delta/t = 0.5$ in the right one. Perturbation theory provides a reliable estimate of the ground state energy per particle, even for $V/t = 5$ where the relative error is approximately 2%.

which is diagonal up to order t/V. The effective Hamiltonian including corrections to order t^3/V^2 is then obtained projecting $\bar{H}^{(3)}$ in the low-energy manifold with $M = zp$ n.n. occupied sites. By performing, long but straightforward calculations we conclude:

$$\mathcal{H}^{(p)} = H_{zp} - \sum_{q=1,2} \frac{T^\dagger_{q,zp} T_{q,zp}}{qV} + \sum_{q=1,2} \frac{T^\dagger_{q,zp} T_{0,zp+q} T_{q,zp}}{(qV)^2} - \frac{1}{2} \sum_{q=1}^{2} \frac{\{T^\dagger_{q,zp} T_{q,zp}, T_{0,zp}\}}{(qV)^2}, \tag{S73}$$

where $H_{zp} = H_0 + T_{0,zp}$ valid up to $\mathcal{O}(t^4/V^3)$.

b. Filling $N_e = N_s$ ($\nu = 1$)

In the $V \to \infty$ limit, the ground state is determined by minimizing the Coulomb energy, $V \sum_{\langle r,r' \rangle} n_r n_{r'}$. This corresponds to the sublattice polarized insulator:

$$|\Phi_0\rangle = \prod_{r \in A} f^\dagger_r |0\rangle, \tag{S74}$$

belonging to the zero momentum sector $Q = \Gamma$. The first order correction in t/V to the ground state energy reads:

$$\delta E_0 = \sum_n \frac{\langle \Phi_0 | T_{-2} | \Phi_{0,n2} \rangle \langle \Phi_{0,n2} | T_2 | \Phi_0 \rangle}{E^{(0)}_0 - E^{(0)}_{0,n2}}, \tag{S75}$$

where $|\Phi_{0,\gamma,2}\rangle$ is obtained by acting with T_2 on the fully sublattice polarized ground state $|\Phi_0\rangle$. Thus, the excited configuration corresponds to a dipole, with energy $\Delta + 2V$, situated along the bond connecting r and $r + u_j$. In this case, the excited subspace is localized and the projected hopping $-t\mathbb{P}_2 \sum_{\langle r,r' \rangle} f^\dagger_r f_{r'} \mathbb{P}_2$ in the subspace of 2 n.n. occupied sites at filling $\nu = 1$ is trivial and does not introduce any quantum dynamics. The energy correction reads:

$$\delta E_0 = -N \frac{3t^2}{\Delta + 2V}, \tag{S76}$$

where 3 is the coordination number of the honeycomb lattice. Fig. S20 shows the comparison between the t/V perturbation theory result and the ground state energy obtained from ED.

c. charge-e excitation

In the strong coupling limit $V \to \infty$, adding an extra carrier incurs an energy cost of $E_{1e} = \Delta + 3V$ with an infinite effective mass, thus, to leading order, realizing a charge-e quasiparticle in a perfectly flat band. The effective hopping

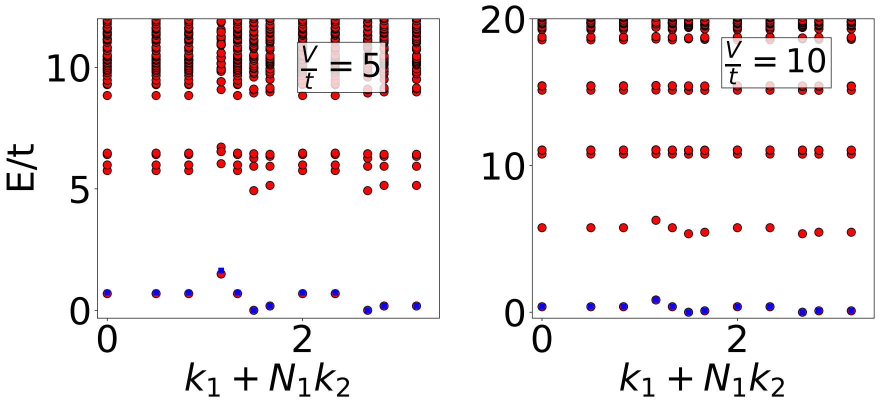

FIG. S21. Many-body spectrum for the charge sector with $p = 1$ doped charge excitation for $\Delta/t = 0.5$. The lowest energy branch of the many-body spectrum describes the charge-e excitation. Blue square data shows the analytical prediction for the dispersion relation obtained order t/V in perturbation theory.

of the charge-e excitation involves an intermediate polaron excitation generated by T_1 and leads to the hopping Hamiltonian:

$$H_f = t_f \sum_{\langle r,r' \rangle \in B} f_r^\dagger f_{r'}, \quad t_f = \frac{t^2}{\Delta + V}. \tag{S77}$$

The latter describes particles hopping on a triangular lattice with dispersion relation $\epsilon_k = 2t_f \sum_{j=1}^{3} \cos(k \cdot a_j)$ displaying minima at $k = K, K'$ and effective mass $m_f = 2/(3t_f) \sim V$.

We now derive the first order correction to the ground state energy in t/V. To this aim, we consider the wavefunction with total momentum K:

$$|\Phi_1\rangle = \frac{1}{\sqrt{N}} \sum_{r \in A} f_{r+u_1}^\dagger e^{-iK\cdot(r+u_1)} |\Phi_0\rangle, \tag{S78}$$

where $K = (2g_1 + g_2)/3$ and $g_{1/2}$ reciprocal lattice vectors. For each configuration $|\Phi_1(r)\rangle$, there are two different hopping processes: those involving the three sites r, $r + a_1$ and $r + a_6$ nearest-neighbors of the B-sublattice occupied site $r + u_1$ and the sites r' away from this region. The second order energy correction originating from processes away from these sites is:

$$\delta E_{1,1} = -(N-3)\frac{3t^2}{\Delta + 2V}. \tag{S79}$$

The contribution of the remaining sites $\{r, r + a_1, r + a_6\}$ is given by:

$$\delta E_{1,2} = -3\frac{2t^2}{\Delta + V} + \epsilon_{K/K'} = -\frac{9t^2}{\Delta + V}. \tag{S80}$$

Taking for simplicity the site r, the first contribution arises from the action of $-t f_{r+u_{2/3}}^\dagger f_r$, which creates a polaron that subsequently recombines without involving any motion of the additional doped carrier at r. Conversely, the second contribution represents the kinetic energy gain from adding the extra carrier at K. Notably, this correction can be explicitly determined by considering second-order processes that involve the motion of the doped carrier. The ground state energy to first order in t/V reads:

$$E_1 = \Delta + 3V - (N-3)\frac{3t^2}{\Delta + 2V} - \frac{9t^2}{\Delta + V}. \tag{S81}$$

Thus, we conclude that the lowest energy of a charge-e quasiparticle up to first order in t/V is:

$$E_{1e} = E_1 - E_0 = \Delta + 3V - \frac{9t^2}{\Delta + V} + \frac{9t^2}{\Delta + 2V} = \Delta + 3V - \frac{9t^2 V}{(\Delta + V)(\Delta + 2V)}. \tag{S82}$$

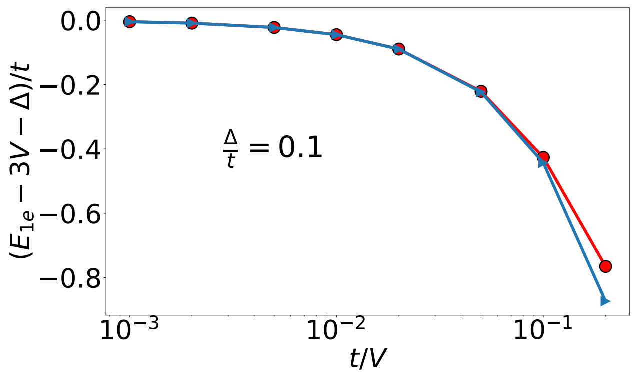

FIG. S22. Energy of a charge-e excitation measured with respect to the charging energy $\Delta + 3V$. Reducing V increases the difference with respect to the perturbative result. The maximum relative error is 14% for $V/t = 5$ and becomes negligible (smaller than 1%) for $V/t \geq 20$.

Fig. S22 shows the comparison between the energy of a single doped excitation obtained with ED (red data) and the result of perturbation theory both measured with respect to the charging energy $\Delta + 3V$.

d. charge-e polaron excitation

In this section we characterize the properties of a charge-e excitation coupled to a single dipole. This excited state has energy $\Delta + V$ above the charge-e quasiparticle. Interestingly, the basis of many-body states spanning the manifold is composed by three different configurations classified by their different eigenvalues under C_{3z}:

$$|\Phi_{1,\ell}(r)\rangle = \frac{1}{\sqrt{3}} \sum_{j=1}^{3} \omega^{\ell(j-1)} f^\dagger_{r+u_j} f^\dagger_{r+u_{j+1}} f_r \, |\Phi_0\rangle \, , \tag{S83}$$

where $\omega = \exp(2\pi i/3)$ and $\ell = 0, \pm$. These states form an orthonormal basis and features three different eigenvalues under C_{3z}. Up to order t/V the Hamiltonian in this subspace reads:

$$\mathcal{H}^{(1)}_{\text{polaron}} = \Delta + V + \frac{T^\dagger_{-1,4} T_{-1,4}}{V + \Delta} - \sum_{q=1,2} \frac{T^\dagger_{q,4} T_{q,4}}{(qV + \Delta)}, \tag{S84}$$

where the energy is measured with respect the charge-e unperturbed energy $\Delta + 3V$. The first contribution $q = -1$ corresponds to the process of recombination of the electron-hole excitation. This process does not induce any dispersion but produces an energy splitting, in the basis $[|\Phi_{1,0}(r)\rangle \, , |\Phi_{1,+}(r)\rangle \, , |\Phi_{1,-}(r)\rangle]^T$ we have:

$$\frac{T^\dagger_{-1,4} T_{-1,4}}{V + \Delta} = \frac{t^2}{V + \Delta} \begin{pmatrix} 0 & 0 & 0 \\ 0 & 3 & 0 \\ 0 & 0 & 3 \end{pmatrix} . \tag{S85}$$

On the other hand, $T^\dagger_{1,4} T_{1,4}/(V)$ induces hopping in real space. Before moving on, it is interesting to notice that:

$$\langle \Phi_{1,a}(r + a_j) | T^\dagger_{1,4} T_{1,4} | \Phi_{1,0}(r) \rangle \, /V = 0, \, \forall j, a \tag{S86}$$

implying that the configuration is localized due to a destructive interference effect. On the other hand, $|\Phi_{1,\pm}(r)\rangle$ develops a finite dispersion relation.

$$\mathcal{H}^{(1)}_{\text{polaron}}(k) = \Delta + V + \frac{t^2}{\Delta + V} \begin{pmatrix} 0 & 0 & 0 \\ 0 & 3 + \sum_{j=1}^{3} \cos(k \cdot a_j + \pi/3) & \sum_{j=1}^{3} \cos k \cdot a_j \\ 0 & \sum_{j=1}^{3} \cos k \cdot a_j & 3 + \sum_{j=1}^{3} \cos(k \cdot a_j - \pi/3) \end{pmatrix} . \tag{S87}$$

We found that the minimum of the dispersion is located at K/K'.

e. Perturbative corrections to the charge gap

In this section, we discuss the renormalization of the charge gap due to perturbative corrections.

To begin, we note that, to leading order in t/V, the energy of an isolated hole is $E_{-1} = 0$. Including first order corrections, up to order t^3/V^2 we obtain:

$$E_{-1} = -(N-2)\frac{3t^2}{\Delta + 2V} - \frac{9t^2}{\Delta + V}. \tag{S88}$$

Finally, the charge gap reads:

$$E_{\text{gap}} = E_1 + E_{-1} - 2E_0 = \Delta + 3V - 3t^2\frac{\Delta + 7V}{(\Delta + V)(\Delta + 2V)}. \tag{S89}$$

Thus, perturbative corrections in t/V reduce the charge gap size. Within perturbation theory the gap collapse at $V = 1.87t$, overestimating the actual critical value of $1.3t$ displayed in Fig. S10d).

f. charge-2e excitation

In the $V \to \infty$ limit, a pair of e excitations in a sublattice-polarized ground state binds to form an excitonic Cooper pair, represented by the bosonic quasiparticle b_r. As discussed previously, the state $|\Phi_2(r)\rangle = b_r^\dagger |\Phi_0\rangle$ is an eigenstate of $H_{M=6}$ (S56) with energy $E_A + 2\Delta + 6V$ and E_A given in Eq. (S41). To leading order in t/V has infinite effective mass resulting in a perfectly flatband of excitonic Cooper pairs. In the following we derive the effective mass, the ground state energy E_{N+2} and the binding energy E_b to first order in t/V.

g. charge-2e dispersion relation

To order t/V the effective hopping of the $2e$-excitation is determined performing degenerate perturbation theory in the low-energy manifold spanned by the basis of states $|\Phi_2(r)\rangle = b_r^\dagger |\Phi_0\rangle$ with $\langle \Phi_2(r)|\Phi_2(r')\rangle = \delta_{r,r'}$. To leading order, the action of H' (S57) induces an excitation with energy scaling as qV (with $q = 1$ for a polaron excitation and $q = 2$ for a dipole [9, 19]). At a subsequent stage, this excitation recombines, connecting a Cooper pair initially located at r to a Cooper pair at $r + a_j$. The resulting nearest-neighbor tunneling amplitude reads:

$$t_b = \sum_{q=1,2} \langle \Phi_2(r + a_j)| \left(T_{-q} \frac{1}{H_{q+6} - E_2^{(0)}} T_q \right) |\Phi_2(r)\rangle \,, \tag{S90}$$

where $E_2^{(0)}$ is the unperturbed ground state energy for two doped carriers, $E_2^{(0)} = E_A + 2\Delta + 6V$. Furthermore, we observe that the amplitude in Eq. (S90) does not depend on the nearest-neighbor site $r + a_{j=1,\cdots,6}$ as a result of the symmetries of the model. We note that the action of T_q on $|\Phi_2(r)\rangle$ transitions the system to an excited manifold, with an energy increase of qV relative to the lowest-energy subspace $q = 0$. Within this excited manifold, the unperturbed Hamiltonian $H_{M=6+q}$ features a non-trivial quantum dynamics with spectrum:

$$H_{M=6+q} |\Phi_{2,nq}\rangle = (qV + E_{2,nq}^{(0)}) |\Phi_{2,nq}\rangle \,, \tag{S91}$$

where $\Phi_{2,nq}$ is the eigenstate n in the subspace of q extra nearest-neighbor occupied sites and $E_{2,nq}^{(0)}$ the corresponding eigenvalues where the superscript refer to the fact that it is computed with respect to the unperturbed Hamiltonian $H_{M=6+q}$. Expanding the resolvent $1/(H_{6+q} - E_2^{(0)})$ in the manifold of eigenstates (S91) we find:

$$t_b = \sum_{q=1,2} \sum_n \frac{\langle \Phi_2(r + a_j)|T_{-q}|\Phi_{2,nq}\rangle \; \langle \Phi_{2,nq}|T_q|\Phi_2(r)\rangle}{qV + E_{2,nq}^{(0)} + 2E_b}, \tag{S92}$$

generalizing the result presented in the maintext to arbitrary V/Δ. We conclude that the dispersion of the Cooper pair results from the constrained quantum dynamics generated by $H_{M=6+q}$ within the excited energy manifold. We now fix a pair of sites r and $r + a_6$, our task is to determine all the possible second order processes connecting two Cooper pairs. By listing these processes we find that to second order only an intermediate polaron T_1 connects the

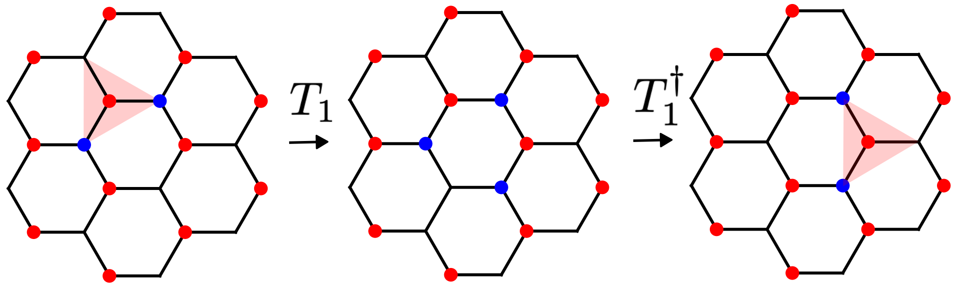

FIG. S23. Motion of a Cooper pair from r to $r + a_6$ creating a polaron at $r + a_5$ along the bond u_1. The intermediate configuration is connected through $H_0 + T_0$ to many other states which are not shown.

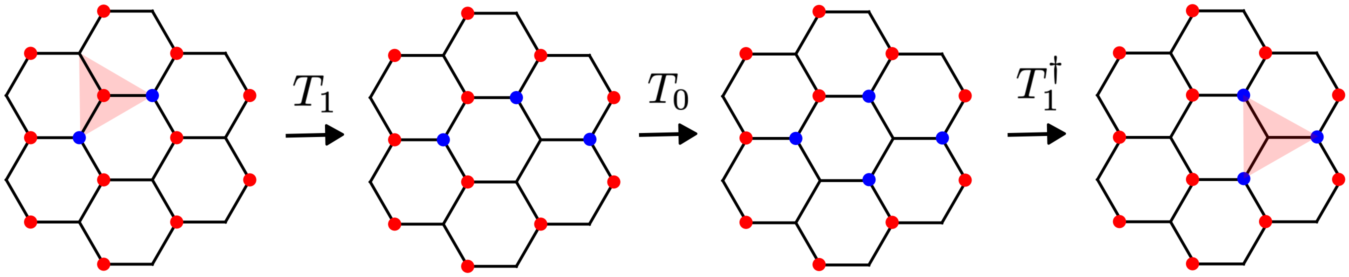

FIG. S24. Motion of a Cooper pair from r to $r + a_6$ creating a polaron at $r + a_6$ along the bond u_1. The intermediate action of T_0 is required in order to connect with the final state.

two configurations. In general, there are many independent paths connecting the initial and final configurations, two of them are displayed in Fig .S23 and in Fig. S24. The first one connects $|\Phi_2(r)\rangle$ to $|\Phi_2(r + a_6)\rangle$ exciting a polaron along the bond $r + a_5$ to $r + a_5 + u_1$. The second one, instead, is characterized by the formation of a polaron along the bond $r + a_6$ to $r + a_6 + u_1$ and requires the intermediate action of $T_{q=0}$ leading to an hopping which does not change the number of n.n. occupied sites. Including all possible processes we find that the dynamics induced by $H_{M=7}$ is composed by 14 configurations.

Fig. S25 shows the dispersion relation in the sector of 2 extra doped particles for different values of V/t and $\Delta/t = 0.5$. Increasing V/t, our perturbative result offers an increasingly accurate approximation of the dispersion

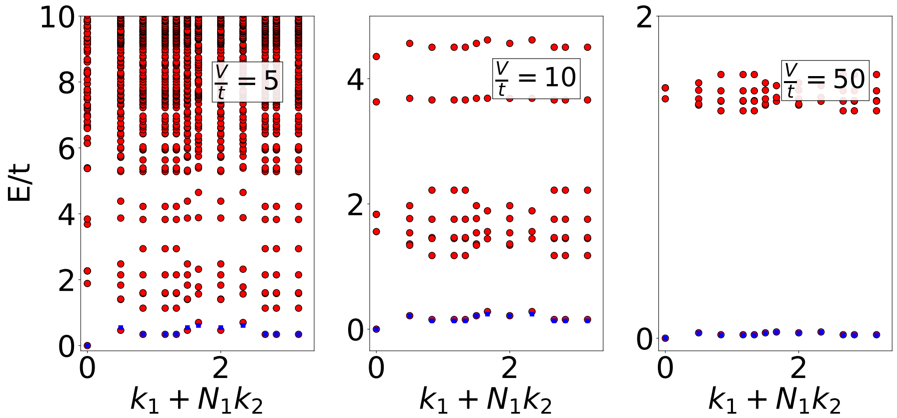

FIG. S25. Many-body spectrum for the charge sector with $N_s + 2$ particles. The lowest energy band of excitations describes the $2e$ exciton Cooper pair. Blue square data shows the analytical prediction $u_b(\mathbf{q}) = -2t_b \sum_{j=1}^{3} \cos(\mathbf{q} \cdot \mathbf{a}_j)$ for the dispersion relation obtained in perturbation theory. The maximum relative error for the different values of V/t are 17%, 12% and 2% for $V/t = 5, 10, 50$, respectively. We employed $\Delta/t = 0.5$.

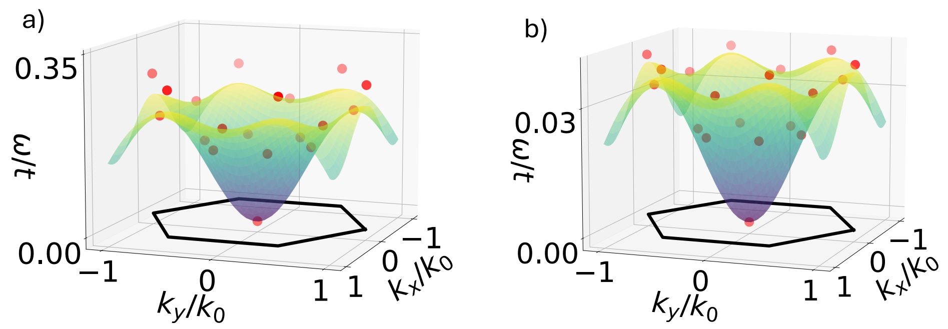

FIG. S26. Excitonic Cooper pair dispersion relation for $V/t = 10$ (left), $V/t = 50$ (right) and $\Delta/t = 0.5$.

relation. We also show in Fig. S26 the dispersion relation of the excitonic Cooper pair in the first Brillouin zone. The dispersion relation obtained to first order in t/V accurately captures the energy points across the spectrum, with the exception of the high-symmetry points K and K' at the band edges, where a relative error of 12% for $V/t = 10$ is observed.

h. charge-2e: binding energy

We now compute the ground state energy gain originating from background intersublattice fluctuations in the presence of the excitonic Cooper pair. In the $V \to \infty$ limit, the ground state energy in this sector is $E_2^{(0)} = 2\Delta + 6V + E_A$ with E_A energy gain in forming the $2e$ excitonic Cooper pair. We perform perturbation theory starting from the ground state with many-body momentum Γ:

$$|\Phi_2\rangle = \sum_{r\in A} b_r^\dagger |\Phi_0\rangle /\sqrt{N}, \tag{S93}$$

where b_r is a bosonic operator creating an excitonic Cooper pair at r. To compute the first order energy correction we introduce the set of sites $\mathcal{S} = \{r, r + a_{j=1,\cdots,6}\}$ nearest-neighbors of the Cooper pair centered at r. The first order correction to the ground state splits in two contributions. The first one involves virtual processes on sites $r \notin \mathcal{S}$ giving rise to the ground state energy correction:

$$\delta E_{2,1} = -(N_s - 7)\frac{3t^2}{\Delta + 2V}, \tag{S94}$$

where 7 counts the number of sites in the set $\mathcal{S}$. The second term instead involves $r \in \mathcal{S}$ around the Cooper pair:

$$\delta E_{2,2} = t^2 \sum_{q=1,2}\sum_{r',r'',r'''\in\mathcal{S}}\sum_{jl=1}^{3} \frac{\langle \Phi_2(r''')|\mathbb{P}_6 f^\dagger_{r''} f_{r''+u_j}\mathbb{P}_{6+q}|\Phi_{2,nq}\rangle\,\langle \Phi_{2,nq}|\mathbb{P}_{6+q} f^\dagger_{r'+u_l} f_{r'}\mathbb{P}_6|\Phi_2(r)\rangle}{E_0^{(0)} - E_{2,nq}^{(0)} - qV}, \tag{S95}$$

where $\mathbb{P}_6$ projects in the subspace with 6 n.n. occupied sites defining the lowest energy subspace for two doped charges while $\mathbb{P}_{6+q}$ projects in the subspace with $q = 1, 2$ extra $\sum_{\langle r,r'\rangle} n_r n_{r'}$. Thanks to the three-fold rotational symmetry and the C_{2x} symmetry we compute the contribution from only one, say $r + a_5$, of the six neighbors in the set $\mathcal{S}$.

Several cases arise: the first one (A) involves the hopping operator $-t f^\dagger_{r+a_5+u_2} f_{r+a_5}$ which acts on the ground state $|r\rangle$ only when the configuration $f^\dagger_{r+u_1} f^\dagger_{r+u_2}\prod_{r'\in A} f^\dagger_{r'}|0\rangle$ is present, weighted by $\alpha/\sqrt{3}$. The configuration $f^\dagger_{r+u_1} f^\dagger_{r+u_2}\prod_{r'\in A} f^\dagger_{r'}|0\rangle$ also couples to an excited state involving a dipole through the action of $-t f^\dagger_{r+a_5+u_{1/3}} f_{r+a_5}$. Summing over these different contributions we find the energy correction

$$\delta E_{2,2A} = -6\frac{t^2}{\Delta + 2V + 2E_b}\alpha^2. \tag{S96}$$

The second case (B) consists of creating an intermediate polaron configuration which then due to the quantum dynamics introduced by $H_0 + T_0$ can either reconnect to the same site or tunnel the Cooper pair to a different site.

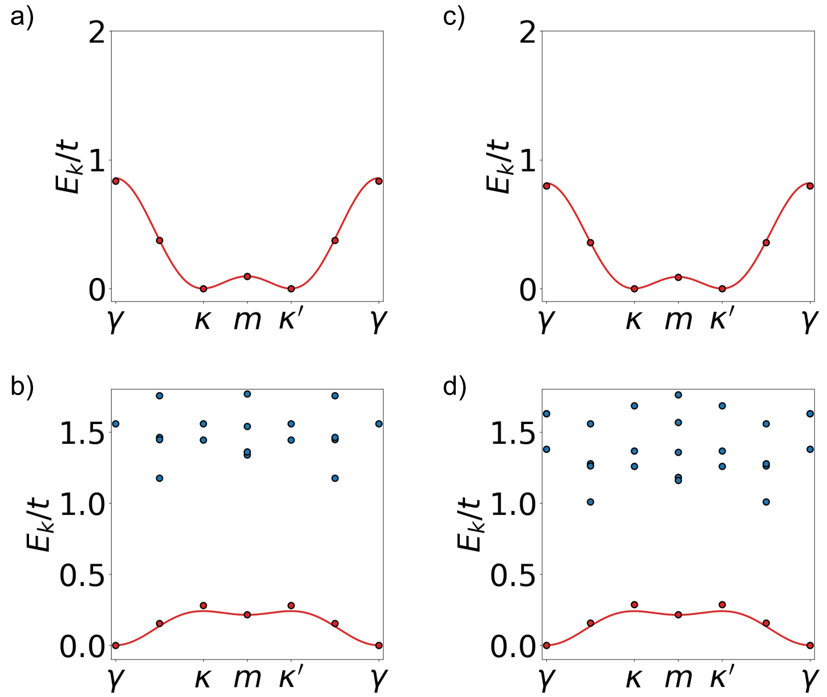

FIG. S27. Panels a)-c) and b)-d) shows the dispersion relation of charge-e and $2e$ excitations, respectively, for $V/t = 10$ and $\Delta/t = 0.5$ (left column) and $\Delta/t = 1$ (right column).

The latter contribution gives an energy gain $-6t_b$ corresponding to the kinetic energy of a Cooper pair at $Q = \Gamma$. The energy variation reads:

$$\delta E_{2,2B} = -6t_b + \sum_{r',r''\in\mathcal{S}} \sum_{jl=1}^{3} \frac{\langle \Phi_2(r)|\mathbb{P}_6 f^\dagger_{r''} f_{r''+u_j}\mathbb{P}_7|\Phi_{2,n1}\rangle \; \langle \Phi_{2,n1}|\mathbb{P}_7 f^\dagger_{r'+u_l} f_{r'}\mathbb{P}_6|\Phi_2(r)\rangle}{E_0^{(0)} - E_{\gamma,1}^{(0)} - V}. \tag{S97}$$

The resulting second-order energy correction is given by the sum $\delta E_2 = \delta E_{2,1} + \delta E_{2,2A} + \delta E_{2,2B}$.

Appendix E: Low-energy effective field theories

In this section, we present analytical results obtained by employing field theoretical approach valid in the "ionic" regime of $\Delta \gg t$.

1. Doping the charge transfer insulator $\Delta/t \gg 1$

In the long wavelength limit, doped carriers are located around K and K' (see Fig. S28) denoted as $\pm$ and their motion is described by:

$$\mathcal{H} = \int \frac{d^2x}{\Omega} \sum_\tau \psi^\dagger_\tau \left(-\frac{\nabla_r^2}{2m_f}\right)\psi_\tau - g\psi^\dagger_+\psi^\dagger_-\psi_-\psi_+, \tag{S98}$$

where ψ_+/ψ_- are Fermi fields for valley K/K', respectively, $m_f = 2/(3t_f)$, $t_f = t^2/(\Delta+V)$ and $g = 6(2\lambda - V_f) = 36t^2V^2/[\Delta(\Delta+V)(\Delta+2V)] > 0$ attractive for arbitrary V and Δ [10]. The problem of two doped carriers reduces to the solution of the Schrödinger equation for the two particle bound state:

$$|\Psi_{2e}\rangle = \int_p F(p)\psi^\dagger_{p+}\psi^\dagger_{-p-}|0\rangle\,, \tag{S99}$$

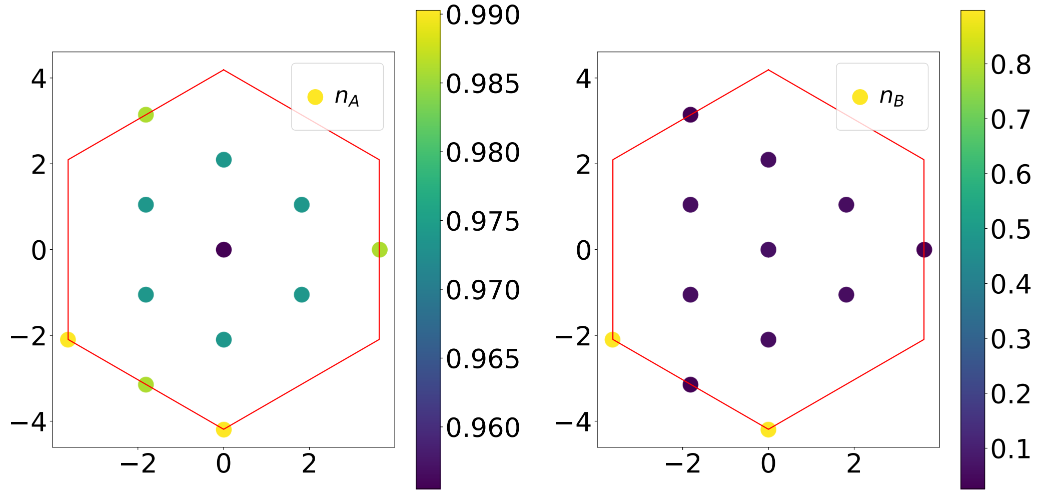

FIG. S28. Momentum space distribution for the two different sublattices for $N_p = 14$ (filling factor $1/2 + 1/12$) also equal to $1/6$ in the B sublattice, $V/t = 10$ and $\Delta/t = 3$. The sum over momenta gives $\sum_k \rho_{k,AA}/N_s \approx 0.978$ and $\sum_k \rho_{k,BB}/N_s \approx 0.189$ ($1/6 = 0.167$ smaller due to intersublattice fluctuations). The color code shows $\langle n_{kA/B}\rangle$.

where the two particle wavefunction $F(p) = F(-p)$ is given by:

$$F(p) = \frac{g}{E_b + p^2/2m_f}, \tag{S100}$$

with E_b binding energy per particle. The binding energy is obtained solving the equation:

$$\frac{1}{g} = \frac{1}{2}\int^{\Lambda} \frac{d^2p}{4\pi^2} \frac{1}{|E_b| + p^2/2m_f} \implies E_b = \frac{\epsilon_\Lambda}{e^{1/\lambda} - 1}, \quad \lambda = \frac{|g| m_f}{4\pi}. \tag{S101}$$

Here, $\epsilon_\Lambda = \Lambda^2/(2m_f)$ is the ultraviolet cutoff, chosen such that E_b agrees with the lattice result in the regime $V \gg \Delta$ and $\Delta \gg t$. Employing Eq. (2) of the main text, we find $E_b \approx 3t^2/(2\Delta)$ which implies $\Lambda = \sqrt{2\pi/3}$ ($a = 1$).

The Cooper pair wavefunction is given by:

$$\Psi_{2e}(\Delta r) = \langle 0|\psi_-(r + \Delta r)\psi_+(r)|\Psi_{2e}\rangle = \int_k F(k) e^{ik\cdot\Delta r}. \tag{S102}$$

The resulting mean square radius reads:

$$\langle r^2\rangle = \frac{\int_r r^2 |\Psi_{2e}(r)|^2}{\int_r |\Psi_{2e}(r)|^2} = \frac{\int_k |\nabla_k F(k)|^2}{\int_k |F(k)|^2} = \frac{1}{3m_f E_b}, \tag{S103}$$

where the last relation follows from Parseval theorem. Finally, the dispersion of the Cooper pairs is obtained expanding the energy of the bound state (S99) for finite center of mass momentum and reads:

$$\frac{1}{m_b} = \frac{1}{2m_f} = \frac{3t^2}{4(\Delta + V)}, \tag{S104}$$

following the same asymptotic behavior as the result obtained in the main text but lacking the correct prefactors.
